# Tumor-infiltrating lymphocytes in HER2-positive breast cancer treated with neoadjuvant chemotherapy and dual HER2-blockade

**DOI:** 10.1038/s41523-024-00636-4

**Published:** 2024-04-18

**Authors:** M. C. Liefaard, A. van der Voort, M. van Seijen, B. Thijssen, J. Sanders, S. Vonk, L. Mittempergher, R. Bhaskaran, L. de Munck, A. E. van Leeuwen-Stok, R. Salgado, H. M. Horlings, E. H. Lips, G. S. Sonke

**Affiliations:** 1 Division of Molecular Pathology, The Netherlands Cancer Institute, Amsterdam, The Netherlands; 2 Department of Medical Oncology, The Netherlands Cancer Institute, Amsterdam, The Netherlands; 3 Division of Molecular Carcinogenesis, Netherlands Cancer Institute, Amsterdam, The Netherlands; 4 Oncode Institute, Utrecht, The Netherlands; 5 Department of Pathology, The Netherlands Cancer Institute, Amsterdam, The Netherlands; 6 Core Facility Molecular Pathology & Biobanking, The Netherlands Cancer Institute, Amsterdam, The Netherlands; 7 Department of Research and Development, Agendia NV, Amsterdam, The Netherlands; 8 Department of Research and Development, Netherlands Comprehensive Cancer Organisation (IKNL), Utrecht, The Netherlands; 9 Dutch Breast Cancer Research Group, BOOG Study Center, Amsterdam, The Netherlands; 10 Department of Pathology, GZA-ZNA Hospitals, Wilrijk, Antwerp Belgium

**Keywords:** Breast cancer, Prognostic markers, Tumour biomarkers, Predictive markers

## Abstract

Tumor-infiltrating lymphocytes (TILs) have been associated with outcomes in HER2-positive breast cancer patients treated with neoadjuvant chemotherapy and trastuzumab. However, it remains unclear if TILs could be a prognostic and/or predictive biomarker in the context of dual HER2-targeting treatment. In this study, we evaluated the association between TILs and pathological response (pCR) and invasive-disease free survival (IDFS) in 389 patients with stage II-III HER2 positive breast cancer who received neoadjuvant anthracycline-containing or anthracycline-free chemotherapy combined with trastuzumab and pertuzumab in the TRAIN-2 trial. Although no significant association was seen between TILs and pCR, patients with TIL scores ≥60% demonstrated an excellent 3-year IDFS of 100% (95% CI 100–100), regardless of hormone receptor status, nodal stage and attainment of pCR. Additionally, in patients with hormone receptor positive disease, TILs as a continuous variable showed a trend to a positive association with pCR (adjusted Odds Ratio per 10% increase in TILs 1.15, 95% CI 0.99–1.34, *p* = 0.070) and IDFS (adjusted Hazard Ratio per 10% increase in TILs 0.71, 95% CI 0.50–1.01, *p* = 0.058). We found no interactions between TILs and anthracycline treatment. Our results suggest that high TIL scores might be able to identify stage II-III HER2-positive breast cancer patients with a favorable prognosis.

## Introduction

The standard of care for patients with stage II-III HER2-positive breast cancer is to receive preoperative chemotherapy combined with dual HER2-targeting therapy consisting of trastuzumab and pertuzumab^1,2^. While response rates are high and long-term prognosis is excellent with this treatment, there is a clinically unmet need to identify patients who may benefit of intensified or de-escalated treatment regimens. First of all, in the neoadjuvant setting, both anthracycline-containing and anthracycline-free chemotherapy regimens are available, but there is no consensus on how clinicians should decide between these treatments. In the TRAIN-2 and TRYPHAENA trials, it was demonstrated that both pCR and survival outcomes are similar for anthracycline-free and anthracycline-containing chemotherapy regimens^3–6^. Given that the incidence of cardiotoxicity and secondary hematologic malignancies is higher in patients who receive anthracyclines, other preoperative treatment regimens may be preferrable^3^. However, it is undetermined if a subgroup of patients may still benefit from anthracyclines. Second, previous studies have shown that some patients may reach pathological complete response (pCR) and have favorable long-term outcomes following de-escalated regimens consisting of shorter chemotherapy duration or only HER2-blockade without any chemotherapy^7–9^. Reliable identification of patients with a high likelihood of response is pertinent for the success of future clinical trials investigating de-escalation strategies. Lastly, adjuvant treatment is currently based on pCR; patients with residual invasive disease after neoadjuvant treatment are eligible for adjuvant trastuzumab emtansine (T-DM1) whereas patients with pCR receive trastuzumab^10,11^. However, some patients may have a good prognosis despite not reaching pCR, and may not need T-DM1^12^. For these reasons, it is highly relevant to investigate both prognostic as well as predictive biomarkers in early HER2-positive breast cancer. Several clinical and molecular markers have been associated with outcome in previous studies, but thus far none have proven to be practice-changing.

Trastuzumab and pertuzumab are monoclonal antibodies that exert their effect by preventing HER2-pathway activation, and by invoking an immune response, mainly through activation of antibody-dependent cellular cytotoxicity (ADCC)^13–16^. Tumors with high infiltration of immune cells may be more responsive to trastuzumab and pertuzumab. Cytotoxic drugs are also capable of eliciting an immune response; anthracyclines have been associated with immunogenic cell death in mice, and taxane treatment has been shown to lead to enhanced T-cell and natural killer-cell function in breast cancer^17–19^. Previously, it has been shown that tumor-infiltrating lymphocytes are associated with increased pCR rates and improved prognosis in several clinical trials of HER2-positive breast cancer treated with chemotherapy and trastuzumab^20–23^. However, in trials of dual HER2-blockade associations between TILs and outcome have been less homogeneous^24–26^. Moreover, interactions between TILs and anthracycline treatment have not been studied.

The objective of this study was to evaluate the prognostic and predictive value of TILs in patients with HER2-positive breast cancer treated with neoadjuvant chemotherapy with or without anthracyclines combined with trastuzumab and pertuzumab in the TRAIN-2 trial. In addition, we investigated the association between expression of immune genes and gene signatures and outcome.

## Results

### Study population characteristics

TILs could be scored in 389 out of 438 patients (88.9%) of the TRAIN-2 study. For 26 patients no slides of pre-treatment biopsies could be obtained due to limited amounts of archival tumor material being available or local laboratory restrictions on sharing of samples for analyses. For 23 patients scoring was not possible due to low quality of the available H&E slides. Descriptive statistics of the studied population are presented in Table 1, and were representative of the overall TRAIN-2 population^27^. The majority of patients (*n* = 349; 89.7%) had a HER2 IHC score of 3+. The remainder of the patients had HER2-positive disease confirmed by in situ hybridization, including one patient who had a HER2 IHC score of 1+ (Table 1). The median TIL score was 7.1% and scores were skewed with 60.7% of scores being lower than 10% (Supplementary Fig. 1). The concordance correlation coefficient between the TIL scores by two pathologists was 0.74 (95% CI 0.70–0.78, Supplementary Fig. 2). The percentage of patients with a concordant classification was 82.2% for the 14% cut-off and 89.8% for the 60% cut-off (Supplementary Table 1).

**Table 1:** Characteristics of the study population

	FEC-T	PTC	*p*-value*
	(*N* = 193)	(*N* = 196)	
Age (years)			0.982
Mean (SD)	49.2 (9.50)	49.2 (10.5)	
Median [Min, Max]	49.0 [25.0, 73.0]	48.0 [22.0, 74.0]	
HR status			1.000
Negative	76 (39.4%)	77 (39.3%)	
Positive	117 (60.6%)	119 (60.7%)	
Grade			1.000
1-2	99 (51.3%)	100 (51.0%)	
3	86 (44.6%)	87 (44.4%)	
Missing	8 (4.1%)	9 (4.6%)	
cT-stage			0.494
cT0-2	127 (65.8%)	137 (69.9%)	
cT3-4	65 (33.7%)	59 (30.1%)	
Missing	1 (0.5%)	0 (0%)	
cN-stage			0.456
cN0	77 (39.9%)	70 (35.7%)	
cN+	116 (60.1%)	126 (64.3%)	
HER2 IHC			0.409
1-2+	15 (7.8%)	21 (10.7%)	
3+	176 (91.2%)	173 (88.3%)	
Missing	2 (1.0%)	2 (1.0%)	
Histology			0.708
Ductal	173 (89.6%)	178 (90.8%)	
Lobular	9 (4.7%)	6 (3.1%)	
Other	11 (5.7%)	12 (6.1%)	
TILs (%)			0.47
Median [Min, Max]	7.07 [1.00, 85.0]	7.07 [1.00, 92.5]	

*FEC-TP* 5-Fluoruracil, Epirubicin, Cyclophosfamide, Trastuzumab, Pertuzumab, *PTC-P* Paclitaxel, Trastuzumab, Carboplatin, Pertuzumab, *HR status* hormone receptor status, *cN0* clinical node negative, *cN+* clinical node positive, *HER2 IHC* HER2 immunohistochemistry scoring, *TILs* Tumor Infiltrating Lymphocytes, *N* number, *SD* standard deviation, *Min* minimum, *Max* maximum.

*P-values are from chi-square tests for categorical variables, t-test for age and Wilcoxon test for TILs.

### Clinical variables and TILs

HR status and tumor grade were associated with TIL levels (Supplementary Fig. 3a, b). No significant association was observed between TILs and clinical T-stage, clinical N-stage, HER2 immunohistochemistry scores or treatment arm (Supplementary Fig. 3c–f). TILs were higher in tumors of a ductal histology compared to lobular tumors (Supplementary Fig. 3g).

### Tumor-infiltrating lymphocytes and pCR

**Fig. 1:**
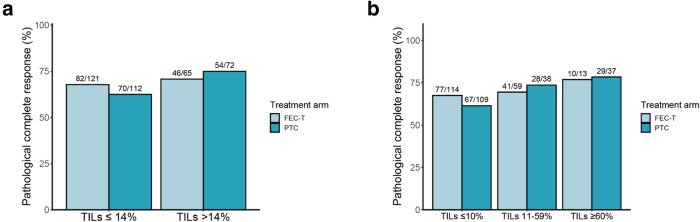
Pathological complete response rate according to TIL scores and treatment. **a** Pathological complete response rate according to treatment arm and TIL score with a 14% cut-off. **b** Pathological complete response rate according to treatment arm and TIL score categorized as low (≤10%), intermediate (11–59%) and high (≥60%). FEC-T 5-Fluoruracil, Epirubicin, Cyclophosphamide, Trastuzumab, Pertuzumab, PTC Paclitaxel, Trastuzumab, Carboplatin, Pertuzumab, TILs tumor-infiltrating lymphocytes.

Plotting of TILs and pCR for the 14% cut-off and in three categories (TILs ≤10% [low]; TILs 11–59% [intermediate], TILs ≥60% [high]), showed a trend towards a positive association between TILs and pCR rate (Fig. 1a, b). In univariable analyses, HR status, HER2 immunohistochemistry score, histological subtype and stromal TILs as a continuous variable were associated with pCR (Supplementary Table 2). TILs as a binary variable were not associated with pCR for both the 14% cut-off as well as for the 60% cut-off. In a multivariable model adjusted for age, HR status, cT, cN, tumor grade, HER2 IHC score and treatment arm, TILs as a continuous variable were no longer significantly associated with pCR (Table 2; Supplementary Table 3). In addition, TILs were not significantly associated with pCR for both the 14% cut-off and the 60% cut-off (Table 2). No interaction was observed between TIL scores and anthracycline treatment (*p* = 0.28 for interaction test with 14% cut-off, *p* = 0.63 for interaction test with 60% cut-off). This was confirmed in analyses of low TIL and high TIL subgroups, performed for both the 14% and the 60% cut-off, which showed no beneficial effect of anthracyclines for any subgroup (Supplementary Tables 4 and 5).

**Table 2:** Multivariable logistic regression analyses with pathological complete response as outcome

	*N*	Event *N*	aOR	95% CI	*p*-value
TILs (continuous, per 10% increase)	349	238	1.09	0.97, 1.23	0.14
TILs					
≤14%	221	145	Ref	Ref	
>14%	128	93	1.19	0.69, 2.06	0.54
TILs					
<60%	301	200	Ref	Ref	
≥60%	48	38	1.87	0.82, 4,58	0.15

*N* Number, *aOR* Adjusted Odds Ratio, *95% CI* 95% Confidence Interval, *Ref* reference, *TILs* Tumor Infiltrating Lymphocytes.

Subgroup analyses based on HR status showed a trend towards an association between continuous TIL scores and pCR in HR positive disease (adjusted Odds Ratio [aOR] 1.15, 95% CI 0.99–1.34, *p* = 0.07; Supplementary Table 6), which was not seen in HR negative disease (aOR 0.99, 95% CI 0.82–1.23, *p* = 0.96; Supplementary Table 6). This trend was also observed when analyzing TILs according to the 60% cut-off (aOR 2.90, 95% CI 1.00–9.71, *p* = 0.063; Supplementary Table 6), but not for the 14% cut-off. In subgroup analyses based on nodal stage, no significant associations were seen between TIL scores and pCR for both node-negative and node-positive disease (Supplementary Table 6).

### Tumor-infiltrating lymphocytes and invasive disease-free survival

**Fig. 2:**
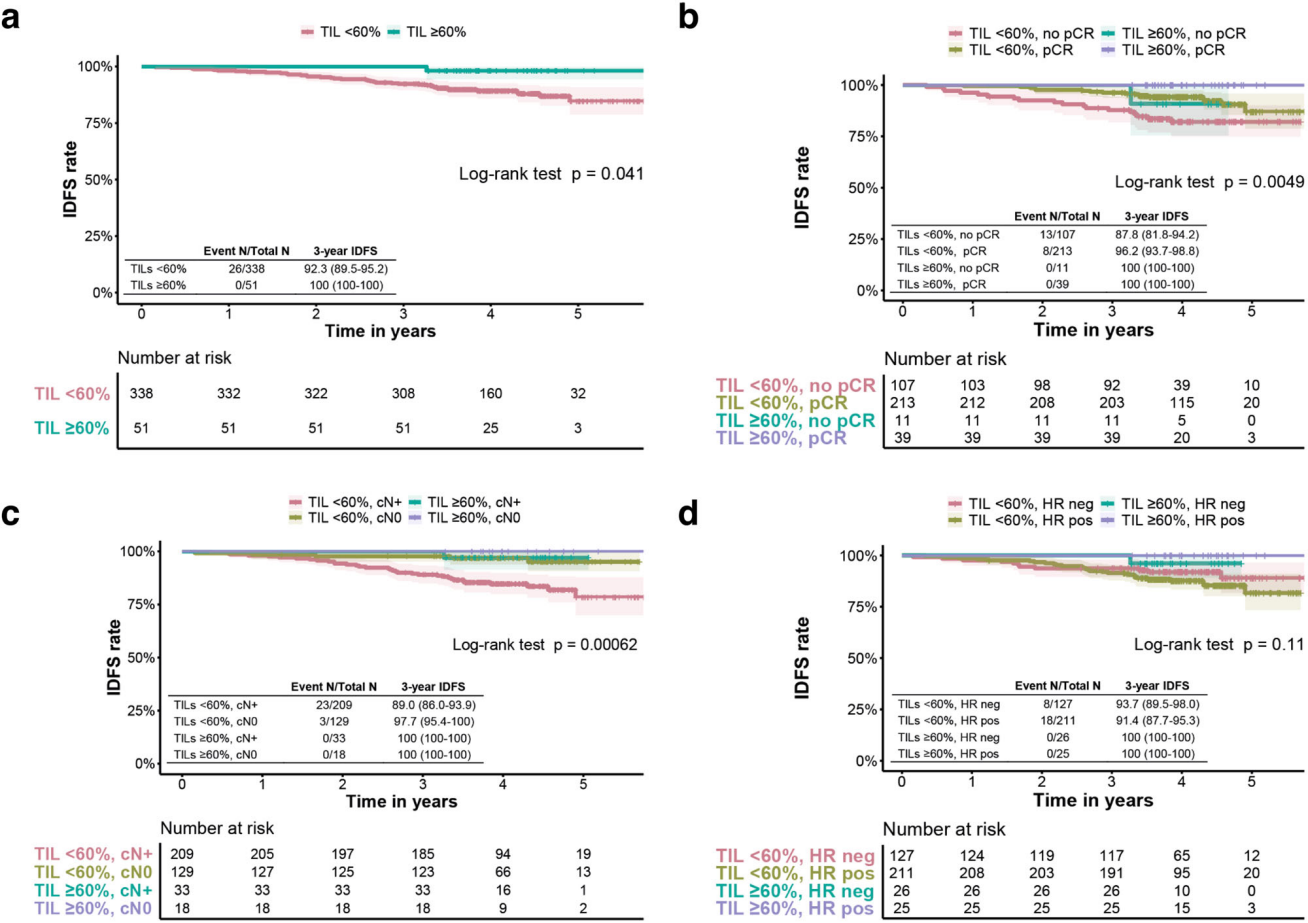
Survival curves according to TILs ≥ 60% and clinical variables. **a** Kaplan–Meier curve for TILs <60% vs. TILs ≥60% at diagnosis. **b** Kaplan–Meier curve for the combination of TILs (60% cut-off) and pCR after neoadjuvant treatment. **c** Kaplan–Meier curve for the combination of TILs (60% cut-off) and nodal stage at diagnosis. **d** Kaplan–Meier curve for the combination of TILs (60% cut-off) and hormone receptor status. All presented *p*-values are from global log-rank tests. TIL tumor infiltrating lymphocytes, IDFS invasive disease free survival, pCR pathological complete response, cN0 node-negative, cN+ node-positive, HR neg hormone receptor negative, HR pos hormone receptor positive.

Median duration of follow-up was 47.7 months (interquartile range 43.0–54.7 months). In patients with TILs ≥60%, IDFS was significantly higher than for patients with TILs <60% (*p* = 0.041; Fig. 2a), with a 3-year IDFS of 100% (95% CI 94.3–100) and 92.3% (95% CI 89.5–95.2) in patients with TILs ≥60% and TILs <60%, respectively. For the 14% cut-off, no significantly different IDFS was seen between the patients with low and high TILs (*p* = 0.22; Supplementary Fig. 4a).

Next, survival analyses were performed for TILs combined with pCR, nodal stage, HR status, and treatment arm, respectively. Kaplan–Meier curves for the combined effect of TILs and pCR on IDFS showed that IDFS rates at 3-years were highest for patients with TILs ≥60% and pCR (100%, 95% CI 100–100; Fig. 2b; Supplementary Table 7). Moreover, IDFS was not significantly different for patients with TILs ≥60% with and without pCR (*p* = 0.119; Supplementary Table 7). In contrast, when evaluating the 14% cut-off in combination with pCR, estimated 3-year IDFS was significantly worse for patients with TILs >14% and no pCR than for those with TILs >14% and pCR (*p* = 0.0076; Supplementary Fig. 4b; Supplementary Table 7).

When evaluating the combined effect of TILs and nodal stage, it was observed that the patients with TILs <60% and node-positive disease had a significantly worse IDFS (3-year IDFS 89.0%, 95% CI 86.0–93.9%) than patients with TILs <60% and node-negative disease (3-year IDFS 97.7%, 95% CI 95.4–100, *p* = 0.0042; Fig. 2c; Supplementary Table 7). In contrast, for patients with TILs ≥60%, no significant difference was seen in IDFS between patients with node-positive and node-negative disease (*p* = 0.55; Supplementary Table 7). Similar results were found when repeating the analysis with the 14% cut-off (Supplementary Fig. 4c; Supplementary Table 7). Kaplan–Meier curves for TILs and HR status showed that although an 8.6% absolute difference in 3-year IDFS for patients with HR positive disease and TIL ≥ 60% versus TIL < 60% was observed, there was no significant difference between the groups for IDFS (*p* = 0.34; Fig. 2d; Supplementary Table 7). Similar results were seen when analyzing the 14% cut-off (Supplementary Fig. 4d; Supplementary Table 7). Lastly, combined analyses of TILs and treatment arm showed no significant difference in IDFS, both for the 14% cut-off and the 60% cut-off (Supplementary Fig. 5e, f; Supplementary Table 7).

Associations between clinicopathological parameters and survival outcomes were further analyzed using Cox regression analyses. In univariable Cox regression analyses, nodal stage, pCR and TILs as a continuous variable were significantly associated with IDFS, whereas other clinical variables were not (Supplementary Table 8). In multivariable analyses correcting for age, grade, tumor stage, nodal stage, HER2 IHC score, and treatment arm, TILs were not significantly associated with IDFS, both when analyzed as a continuous and as a binary variable with a 14%, and 60% (Table 3; Supplementary Table 9). No significant interaction was seen between TILs and treatment arm (*p* = 0.58 for the 14% cut-off, *p* = 0.99 for the 60% cut-off).

**Table 3:** Multivariable Cox regression analyses with invasive disease-free survival as outcome

	*N*	Event *N*	aHR	95% CI	*p*-value
TILs (continuous, per 10% increase)	367	38	0.86	0.70, 1.04	0.12
TILs					
≤14%	233	27	Ref	Ref	
>14%	134	11	0.71	0.35, 1.44	0.339
TILs					
<60%	318	37	Ref	Ref	
≥60%	49	1	0.22	0.03, 1.65	0.141

*N* number, *aHR* Adjusted Hazard Ratio, *95% CI* 95% Confidence Interval, *Ref* reference, *TILs* Tumor Infiltrating Lymphocytes.

Additional analyses in low and high TIL subgroups performed for both the 14% and 60% cut-off confirmed that there was no beneficial effect of anthracyclines based on TIL subgroups, although the sample size limited the interpretation of the model in patients with TILs ≥60% (Supplementary Tables 10 and 11). For patients with TILs ≤14%, positive nodal stage at diagnosis was associated with a significantly increased hazard ratio of 10.7 (95% CI 2.43–47.2, *p* = 0.002; Supplementary Table 10), whereas for patients with TILs >14%, nodal stage was not significantly associated with IDFS (adjusted Hazard Ratio [aHR] 1.15, 95% CI 0.30–4.41, *p* = 0.84; Supplementary Table 10). These analyses were repeated in patient with TIL <60% and ≥60%, but meaningful interpretation was limited due to the small sample size (Supplementary Table 11).

Further subgroup analyses were performed based on clinical variables. In patients with HR positive disease, TILs as a continuous variable showed a trend towards an positive association with IDFS (aHR per 10% increase in TILs 0.71, 95% CI 0.50–1.01, *p* = 0.058; Supplementary Table 12), and a significant association between TILs as a binary variable with a cut-off of 14% and IDFS was observed (aHR 0.34, 95% CI 0.12–1.00, *p* = 0.05; Supplementary Table 12). No association between TILs and IDFS was seen in HR negative disease (aHR 1.97, 95% CI 0.61–6.32, *p* = 0.25; Supplementary Table 12). In subgroups based on nodal status no significant associations were seen between TILs as a continuous variable or TILs at a cut-off of 14% and IDFS (Supplementary Table 12). The 60% cut-off was also investigated but the analyses were limited by small sample size (Supplementary Table 12).

### Gene expression analyses

Gene expression data was available for 323 patients with evaluable TILs. TIL scores correlated negatively with expression of ESR1 and positively with all investigated immune genes and gene signatures. No correlation was seen between TIL and ERBB2 expression (Supplementary Fig. 6). ESR1 gene expression correlated negatively with expression of ERBB2 and most immune genes. ERBB2 expression was positively correlated with expression of FOXP3, CTLA4, PD1 and the trastuzumab-related signature.

In univariable analyses, expression of ESR1 was negatively associated with pCR while expression of ERBB2, GZMB, PRF1, PD1 and IFNG was positively associated with pCR after correction for multiple testing (Supplementary Table 13). In multivariable analyses adjusted for age, HR status, grade, cT, cN, HER2 IHC score, TILs and treatment arm, ERBB2 showed a strong positive association with pCR (aOR 11.56, 95% CI 4.98–29.41; Supplementary Table 13). No other genes were significantly associated with pCR in multivariable analyses. Univariable and multivariable Cox regression analyses showed no significant associations between gene expression and invasive disease free survival (Supplementary Table 14). Subgroup analyses in the two treatment arms demonstrated that in the patients that received FEC-T, ERBB2 expression showed a significant positive association with pCR in multivariable logistic regression analyses. In addition, expression of FOXP3, the TLS-related gene signature and expanded immune gene signature were positively associated with improved IDFS (Supplementary Table 15). In the subgroup that was treated with PTC, no associations between gene expression and outcomes were found, apart from a positive association between ERBB2 expression and pCR (Supplementary Table 16).

## Discussion

In this TRAIN-2 sub study, continuous TIL scores and immune gene expression were not associated with pCR or IDFS, and findings from the TRYPHAENA study with a 14% cut-off for TILs could not be validated for the identification of prognostic subgroups. However, a trend towards increased odds of pCR was observed for a 60% cut-off. Moreover, patients with TILs ≥60% demonstrated excellent 3-year IDFS, regardless of nodal status, HR status and attainment of pCR. Exploratory analyses indicate that TIL scores might be a relevant prognostic factor in HER2-positive, HR positive disease and thus provides an interesting lead for future research. Lastly, we found no evidence that indicates TILs can be used to identify patients with particular benefit of anthracyclines.

Although several studies in patients with HER2-positive breast cancer treated with neoadjuvant chemotherapy and single HER2-blockade have demonstrated an association between higher TILs and improved chances of pCR and a more favorable prognosis, results have been inconsistent in trials of dual HER2-blockade^20–24,26,28–31^. In the TRYPHAENA trial, patients with stage II and III HER2-positive breast cancer were randomized to anthracycline-containing or anthracycline-free chemotherapy regimens combined with trastuzumab and pertuzumab. TILs were not associated with pCR, but for every 10% increase in TIL, a 25% reduction in the hazard of an event was observed after correction for pCR^26^. In the NeoSphere trial, patients were randomized to 4 different treatment arms, including one arm that combined docetaxel, trastuzumab and pertuzumab. Here, an association was seen between TILs and pCR, but for the definition of pCR only invasive disease in the breast was considered, whereas in our study pCR is defined as absence of invasive disease in both the breast and axilla^24^. Lastly, in the GeparSepto trial, TILs were not associated with pCR in patients with HER2-positive disease that received neoadjuvant chemotherapy combined with trastuzumab and pertuzumab^25^. For both the NeoSphere and the GeparSepto, associations with long-term outcomes were not reported.

In our study we studied TILs as a continuous variable, and as a binary variable with two different two cut-offs. The first cut-off of 14% was selected with the intention to reproduce the analyses from the TRYPHAENA study, for which the population and treatment regimen were similar to the TRAIN-2 study. Secondly, we studied TILs with a cut-off of 60%, as previously it has been demonstrated that this may be a clinically relevant cut-off^32^. Lastly, we studied immune gene expression and outcomes.

In our study, we did not find significant associations with outcome when TILs were studied as a continuous variable or a binary variable with a 14% or a 60% cut-off. In contrast to the TRYPHAENA study, our analyses did not show significant associations between expression of single immune genes or immune gene signatures and outcomes. It is noteworthy that in the TRYPHAENA study, the 14% TIL cut-off was based on the median score in the population, whereas in our study the median TIL score was much lower^26^. The difference in results may be partially explained by our study having a higher proportion of patients with HR positive disease. In addition, it may be that, despite the two studies having overlapping population characteristics and using international TIL scoring guidelines, substantial inter-study heterogeneity in TIL scoring exists.

Despite not showing a significant association with pCR, we did observe excellent IDFS outcomes in patients with TIL scores ≥60%, even for patients with node-positive disease at diagnosis and patients who did not reach pCR after treatment. This may suggest that the relation between TILs and outcome may be more distinct when using a higher TIL cut-off, which is in line with results of recent studies, that suggest that patients with highly infiltrated breast tumors seem to have favorable outcomes^21,32,33^. Given that the number of patients with TILs ≥60% in our study was low, our analyses can only be considered exploratory, and our results need to be validated further in comparable larger-sized cohorts. In addition, in our study we report 3-year IDFS estimates, as the amount of censoring after the 3-year follow-up time point was substantial, leading to uncertainty in the estimates and challenges with interpretability. Prolonged and more complete follow-up data would have provided better insights into the associations between TILs and long-term outcomes. Lastly, to evaluate if TIL scores could be of value in selecting patients that may be sufficiently treated with a shorter duration of neoadjuvant treatment, or who may not require chemotherapy and could be treated with only HER2-blockade, it would be of interest to first validate our results in de-escalation trials such as the TRAIN-3 study.

The lack of a strong and consistent association between continuous TIL scores and outcome in patients treated with dual HER2-blockade may be explained by the mechanism of action of HER2-directed antibodies. Besides inhibition of HER2-mediated signaling, trastuzumab and pertuzumab activate antibody dependent cellular cytotoxicity (ADCC) and antibody dependent cellular phagocytosis (ADCP) by binding to FcgRIIIa-receptors on natural killer cells and macrophages^13–16,34^. Previously, xenograft and cell line studies demonstrated that ADCC activation was either not increased for the combination of trastuzumab and pertuzumab in comparison to each single agent alone, or was saturated with increasing dosage, which suggests that the complementary inhibitory effect on the HER2 pathway may be more significant determinant of response than ADCC^35–37^. Our findings that ERBB2 gene expression is strongly associated with pCR, but expression of immune genes is not, may be supportive of this idea. However, another explanation would be that TILs and immune gene expression in pre-treatment biopsies may not be suitable predictors of potential immune activation, as it has been shown that the immune infiltrate is dynamic and changes during treatment are associated with outcome^38^. Studies evaluating TILs in on-treatment biopsies and in residual disease of patients with no pCR, may help to better understand this dynamic state in HER2-positive disease.

To explore if TIL scoring would add value to established prognostic factors, we performed several subgroup analyses. First, we studied HR status, as it is a well-documented predictor of response following dual HER2-directed treatment^39,40^. Interestingly, in our study we found a trend towards better outcomes with higher TILs in patients with HR positive breast cancer. As prior research suggests that activation of ER signaling provides an escape mechanism to the HER2-pathway inhibition, it could be speculated that response in HR positive breast cancer is more dependent on ADCC by monoclonal antibodies^39^. However, our analyses can only be seen as exploratory, as they were limited by the sample size of the subgroups and no significant association were observed. Further studies are necessary to provide detailed insights into the determinants of response in HER2-positive, HR positive breast cancer. Second, results of our analyses suggest that positive nodal stage may in particular be unfavorable for patients with low TIL scores at baseline. In patients with high TIL scores, both node-negative and node-positive subgroups had excellent 3-year IDFS outcomes. However, these results need to be confirmed in other studies, and re-evaluated with longer follow-up. Lastly, we investigated the combination of TILs and pCR in relation to IDFS, as pCR status is considered as a proxy for long-term outcome, and it is used to inform the decision for adjuvant T-DM1 versus adjuvant trastuzumab^10–12^. Although we found that patients with TILs ≥60% had a 100% 3-year IDFS rate regardless of pCR status after treatment, the subgroup of patient with high TILs and no pCR is too small to make recommendations regarding the use of adjuvant agents such as T-DM1. The results need to be further validated in other cohorts.

In the past few years, several studies have confirmed that anthracycline-free regimens are equally effective and less toxic than anthracycline-containing chemotherapy schedules^3,6,27,41^. In our study, we were unable to identify subgroups based on TILs that may have benefit of receiving anthracyclines. Interestingly, we did find that in the patients treated with anthracyclines, several immune genes are associated with IDFS, whereas this was not the case in patients treated with anthracycline-free chemotherapy. Previously it has been shown that anthracyclines induce immunogenic cell death, which may in part explain these results^17,18^. As it has been shown that efficacy and toxicity of anthracycline-free regimens is favorable, and our results add to the existing evidence that there does not seem to be a subgroup that benefits of anthracyclines, it may be preferred to give anthracycline-free regimens. However, the mechanisms underlying chemosensitivity are still unclear and further comprehensive studies of large cohorts are required before anthracyclines can be abandoned as a treatment option altogether.

In our analyses we found higher FOXP3 gene expression to be associated with better IDFS, which is not in line with existing literature demonstrating that low levels of FOXP3 lymphocytes are associated with improved prognosis^42,43^. However, it has been reported that the prognostic value of FOXP3 depends on other factors, such as molecular subtype, cytotoxic T-cell infiltration, and the localization of FOXP3 protein expression^44,45^. Moreover, FOXP3 expression has been confirmed in both healthy breast cancer tissue as well as breast cancer cells^45^. Based on our data, we cannot confirm the origin of the FOXP3 signal which limits interpretation.

The large sample size, and the randomized design of the initial trial are strengths of our study. TIL scoring showed to be highly concordant between the two pathologists, in particular when a higher TIL cut-off was applied. However, we also found significant differences between scores on an individual patient level. Careful consideration should be taken before implementing TILs as a potential biomarker for de-escalation of treatment in future trials, as there is a risk of misclassification and thus undertreatment, in particular for patients with heterogeneous tumors^46^. We recommend that such trials always involve at least two pathologists for TIL scoring, and re-review by a third pathologist in case of discordance. In addition, the evolving research related to spatial infiltration patterns may impact interpretation of TIL scores in the future^47–49^. In our study we did not assess spatial distribution of TILs or interactions with other cells in the tumor micro-environment. Our study is further limited by lack of on-treatment and post-treatment TIL scores, as well as immunohistochemistry scoring of TIL subsets.

In conclusion, our study shows that in patients with stage II-III HER2-positive disease, patients with baseline TIL scores of 60% and higher show an excellent invasive disease-free survival at 3 years, even those with hormone receptor negative or node-positive disease, and those who did not achieve pCR after neoadjuvant treatment. In addition, TILs showed a trend towards an association with response and prognosis in HER2-positive, HR positive disease, which provides a lead for future studies. TIL scores did not identify a subgroup of patients that have a benefit of anthracycline-based neo-adjuvant chemotherapy. Validation of our findings in other studies is warranted to establish the value of TILs and their optimal cut-off for clinical decision-making in HER2-positive disease.

## Methods

### TRAIN-2 study design

The design and results of the TRAIN-2 study (NCT01996267) have been reported previously^3,27,50^. In brief, 438 patients with stage II-III HER2-positive breast cancer were randomized to receive anthracycline-containing or anthracycline-free neoadjuvant chemotherapy combined with trastuzumab and pertuzumab. All patients underwent surgery, radiotherapy, and adjuvant systemic treatment according to local guidelines. The study was conducted in accordance with Good Clinical Practice guidelines and the declaration of Helsinki. Approval for the study and amendments was obtained from the medical ethics committee at the Netherlands Cancer Institute. All participants signed written informed consent.

### HER2 evaluation

In the TRAIN-2 study, baseline tumor biopsies were evaluated locally in participating centers for HER2 status. The tumor was considered HER2-positive in case of overexpression and/or amplification of HER2 in an invasive component of the core biopsy, defined as >10% of invasive tumor cells showing strong complete circumferential membrane staining (score 3+) and/or HER2 gene amplification defined as ≥6 HER2 gene copies per nucleus by in situ hybridization^51^.

### Tumor-infiltrating lymphocyte scoring

Haematoxylin and eosin (HE) slides and formalin-fixed paraffin-embedded (FFPE) blocks of pre-treatment tumor biopsies of TRAIN-2 patients were obtained retrospectively through the nationwide network and registry of histo- and cytopathology in the Netherlands (PALGA)^52^. For patients with missing data, tumor grade was scored by a pathologist (J.S.) using an online platform^53^. Stromal TIL percentages were assessed according to the 2014 guidelines developed by the International Immuno-Oncology Biomarker Working Group^54^. Each case was scored by two out of three independent pathologists (M.v.S., H.H., R.S.) who were not informed of treatment arm and outcome. For cases with more than 10% absolute difference in scores, the slide was reviewed and a final call was made by one pathologist (R.S.). For cases with 10% or less absolute difference, geometric means were calculated after adding 0.5 to values equal to 0. The concordance correlation coefficient was calculated to assess agreement between the two pathologists.

### Gene expression

Tumor cell percentage (TCP) was scored on HE slides for all patients by a pathologist (J.S.). For tumors with a TCP of >30% and sufficient tumor tissue available, ten 5-μm blank slides were sent to Agendia NV for RNA isolation. RNA was isolated using the RNeasy FFPE kit (Qiagen, Valencia, CA) in accordance with the manufacturer’s instructions as previously reported, followed by whole transcriptome sequencing according to an internal pipeline^55,56^. Libraries were prepared with the TruSeq Stranded Total RNA kit (Illumina) and sequenced using a 75 bp single-read protocol on a NextSeq 2000 machine^57^. Gene expression levels were normalized using the trimmed mean of M-values method as implemented in the edgeR package, and transformed to log counts per million. Gene expression scores were calculated for estrogen receptor 1 (ESR1), Erb-B2 Receptor Tyrosine Kinase 2 (ERBB2) and several genes that have been shown to be relevant to outcome or that have been demonstrated to be associated cytotoxic activity: Granzyme A and B (GZMA and GZMB), Perforin 1 (PRF1), Forkhead Box P3 (FOXP3), cytotoxic T-lymphocyte-associated protein (CTLA4), programmed death-ligand 1 (PDL1), programmed cell death protein 1 (PD1) and interferon-gamma (IFNG). In addition, several published gene signatures that previously showed associations with outcome in HER2-positive disease were analyzed: a signature related to STAT1 (immune_STAT1), a signature related to tertiary lymphoid structures (immune_TLS), a signature related to adjuvant trastuzumab benefit (immune_trastuzumab), a 6-gene IFN-γ signature (IFNG_6-gene), an 18-gene expanded immune signature (expanded_immune_18-gene) and a cytolytic activity score^58–62^. For the gene signatures, scores were defined as a weighted sum of the log-expression of the included genes. All individual gene expression scores and signature scores were scaled in the same way as was done in the TRYPHAENA sub study: that is, the 2.5% and 97.5% quantiles were set equal to −1 and +1, respectively. Samples with less than 3 million reads were excluded from the analyses^26^.

### Endpoints

The primary endpoint of the study was pCR, defined as the absence of invasive disease in the breast and lymph nodes (ypTis/0N0). The secondary endpoint, invasive disease-free survival (IDFS), was defined as time from randomization to first documentation of local, loco-regional or distant recurrence of disease, incidence of a new invasive malignancy, or death.

### Statistical analyses

Univariable and multivariable binary logistic regression and Cox regression were performed to assess the relation of clinicopathological variables and TILs with pCR and IDFS, respectively. TILs were analyzed both as a continuous and a binary variable, for which two cut-offs were selected. First, TILs were evaluated with a cut-off of 14%, in line with the intention to reproduce previously published analyses from the TRYPHAENA study^26^. In addition, consistent with a previous publication by Denkert et al., tumors were categorized into low (≤10%), intermediate (11–59%) and high TIL (≥60%), where low and intermediate were grouped into one category for analyses, as the intermediate group was small, and the 60% cut-off has shown most clinical relevance in previous TIL studies^32^. Multivariable analyses were corrected for variables that were found to be significantly associated with pCR or IDFS in univariable analyses and/or for variables that are known from literature to be associated with outcome, including age, HR status, tumor grade, cT-stage, cN-stage and HER2 IHC score, in order to evaluate the independent prognostic value of TILs. To investigate if TILs were predictive of anthracycline benefit, interaction terms of TILs and anthracycline treatment were added to the models, and subgroup analyses were done in low and high TIL subgroups according to the abovementioned cut-offs of 14% and 60%. In addition, the association of TILs and outcome was evaluated in subgroups based on hormone receptor (HR) status and nodal status, given that these variables are known to be relevant for response to neoadjuvant treatment and for prognosis.

The correlation between individual genes, gene signatures and TILs was assessed by Spearman’s correlation coefficient. The association between gene expression scores and pCR was evaluated using univariable and multivariable logistic regression, correcting for the same set of variables as in above-mentioned analyses, and for TIL scores. In addition, gene expression scores and IDFS were analyzed in univariable and multivariable Cox regression models. *P*-values from gene expression analyses were corrected for multiple testing by FDR. All analyses were conducted with R version 4.2.1^63^. Statistical test were two-sided and *p* values < 0.05 were considered significant.

### Reporting summary

Further information on research design is available in the Nature Research Reporting Summary linked to this article.

### Supplementary information

Supplementary Figures and Tables

Reporting summary

## Data Availability

The clinical data and TIL dataset generated during and/or analyzed during the current study are available from the corresponding author on reasonable request. The raw RNAseq data for this study were generated at Agendia NV. This data is available upon reasonable request from the corresponding author with the permission of Agendia NV.

## Code availability

Data analysis was performed with R version 4.2.1. The code used in the study is available from the corresponding author on reasonable request.
