# Supplementary material for: Tumor-infiltrating lymphocytes in HER2-positive breast cancer treated with neoadjuvant chemotherapy and dual HER2-blockade
Source: NPJ Breast Cancer. 2024 Apr 18;10:29. doi: 10.1038/s41523-024-00636-4 (PMC11026378; doi:10.1038/s41523-024-00636-4)
Supplement: Supplementary file 1 — Supplementary Figures and Tables [file 41523_2024_636_MOESM1_ESM.pdf]

SUPPLEMENTARY FIGURES

Supplementary Figure 1. Histogram of TIL scores

Abbreviation: TIL = tumor infiltrating lymphocytes

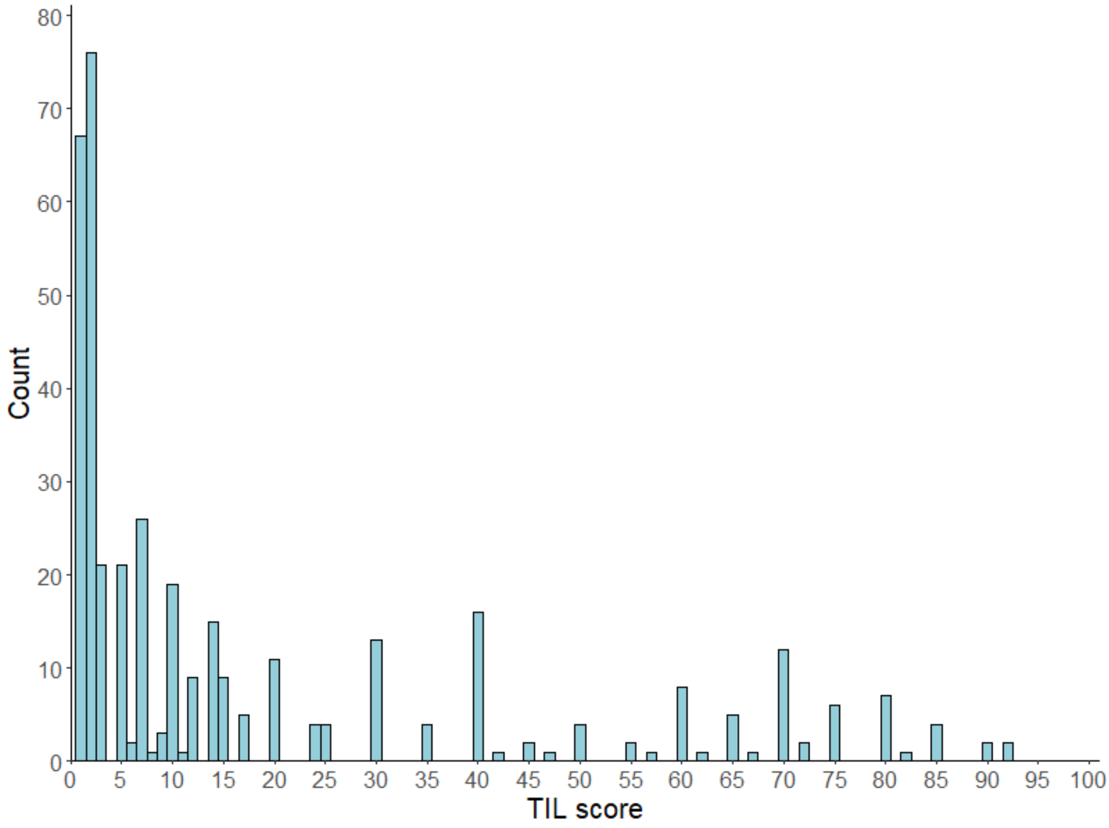

Supplementary Figure 2. Bland-Altman plot of mean TIL scores and difference in TIL scores between the two pathologists

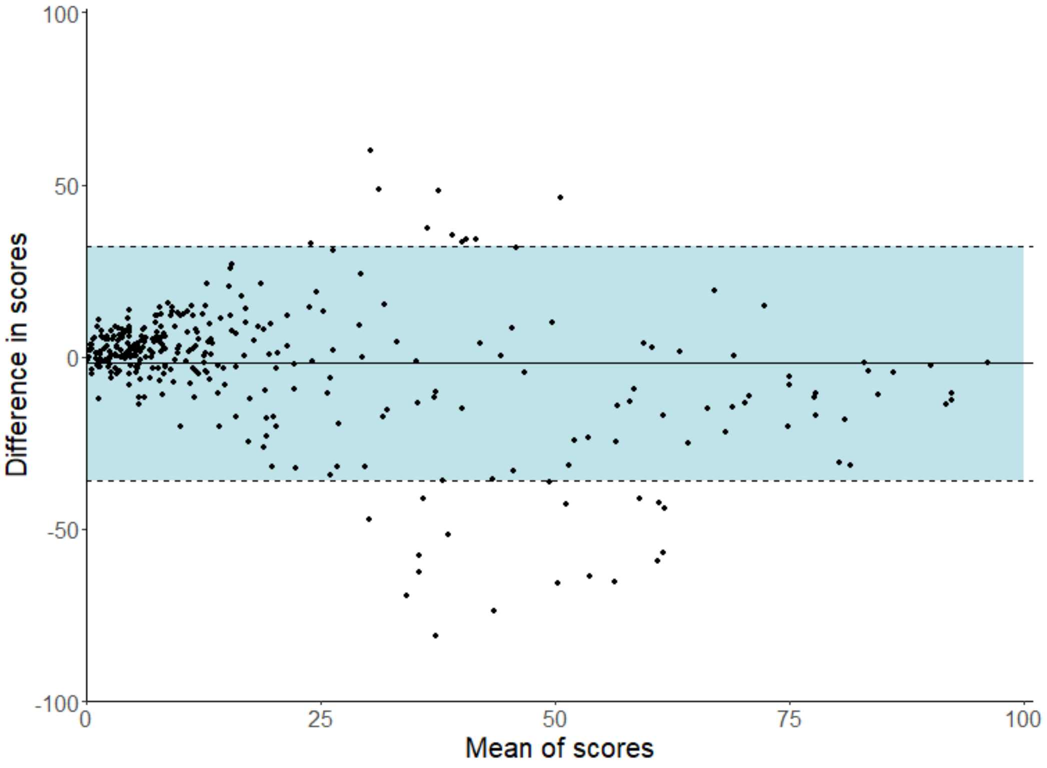

Supplementary Figure 3. Associations of clinical variables and TIL levels

a) Association of hormone receptor status with TIL levels. b) Association of tumor grade with TIL levels. c) Association of clinical T-stage with TIL levels. d) Association of clinical N-stage with TIL levels. e) Association of HER2 IHC score with TIL levels. f) Association of treatment arm with TIL levels. g) Association of histological subtype with TIL levels.
Abbreviations: HR = hormone receptor status; cT = clinical T-stage; cN0 = clinical node-negative; cN+ = clinical node-positive; IHC = immunohistochemistry score; FEC-T = 5-Fluoruracil, Epirubicin, Cyclophosphamide, Trastuzumab, Pertuzumab; PTC = Paclitaxel, Trastuzumab, Carboplatin, Pertuzumab

a

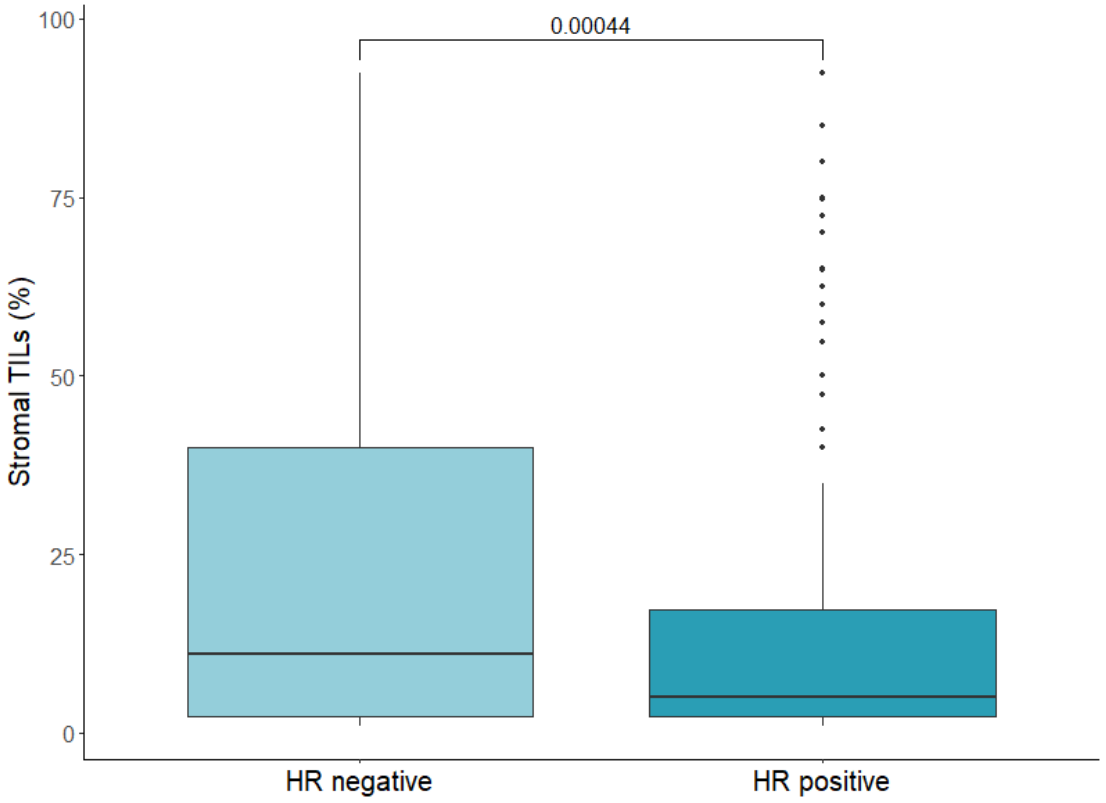

b

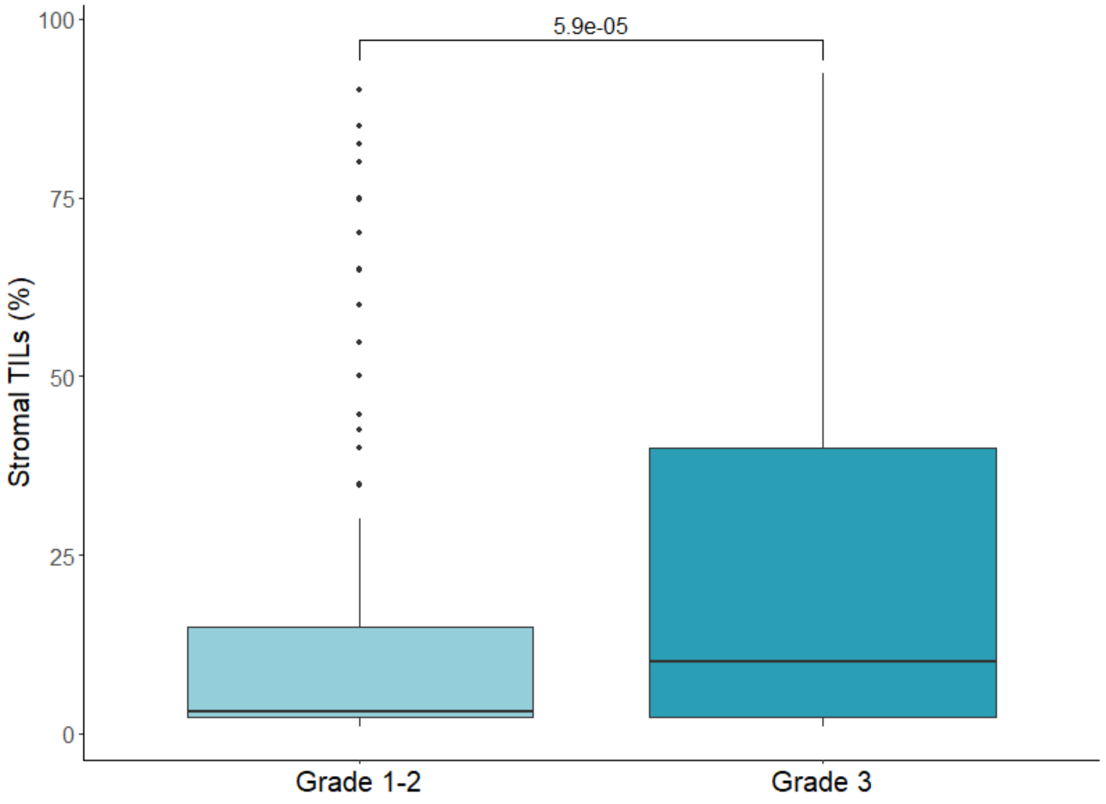
5.9e-05
Stromal TILs (%)
100
75
50
25
0
Grade 1-2
Grade 3

c

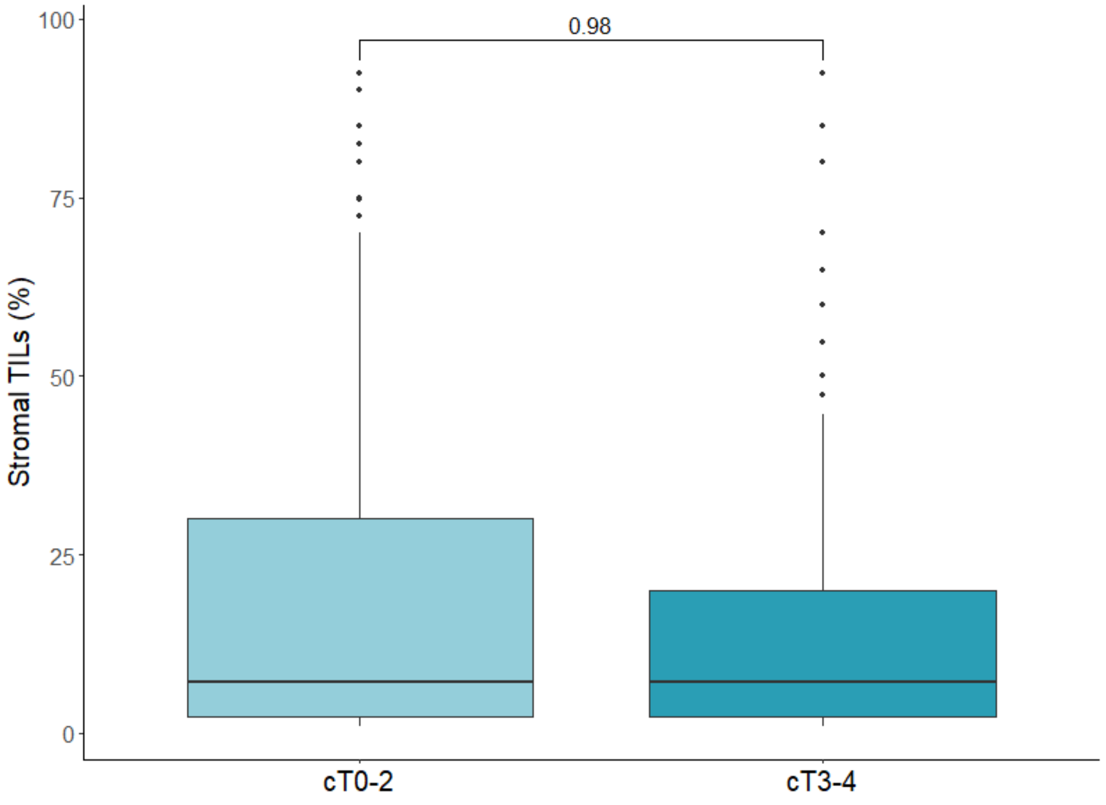
0.98
Stromal TILs (%)
100
75
50
25
0
cT0-2
cT3-4

d

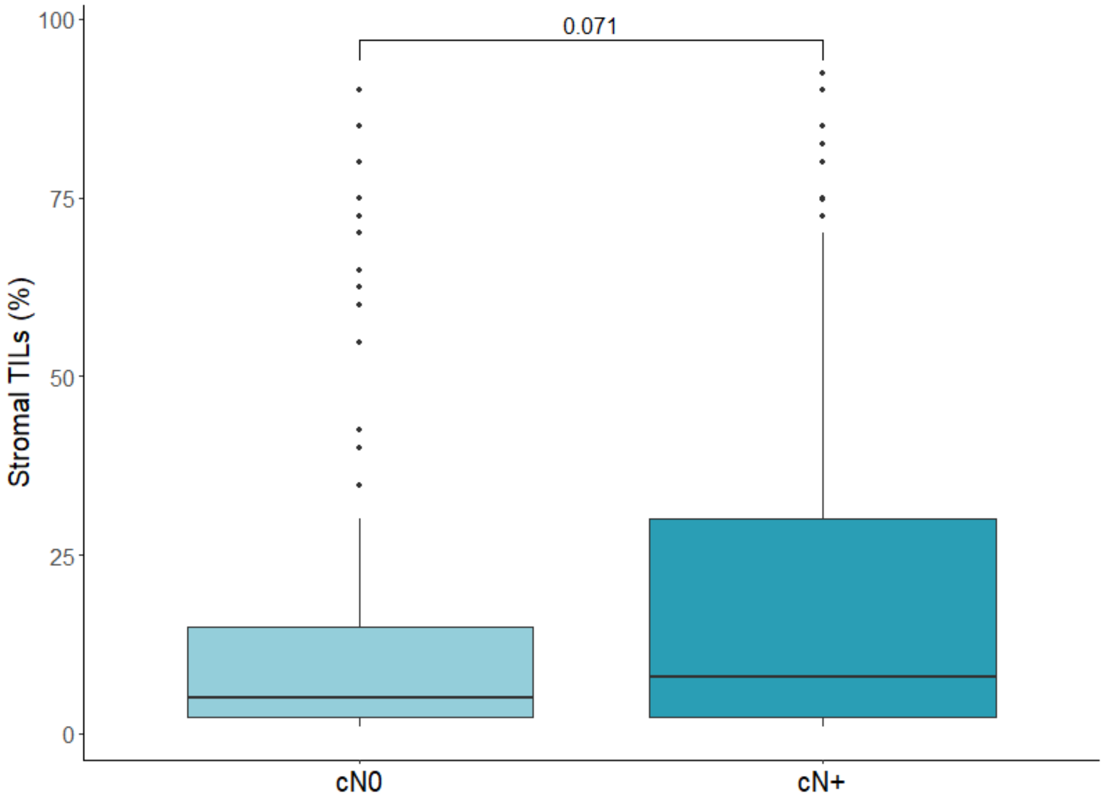

0.071
Stromal TILs (%)
100
75
50
25
0
cN0
cN+

e

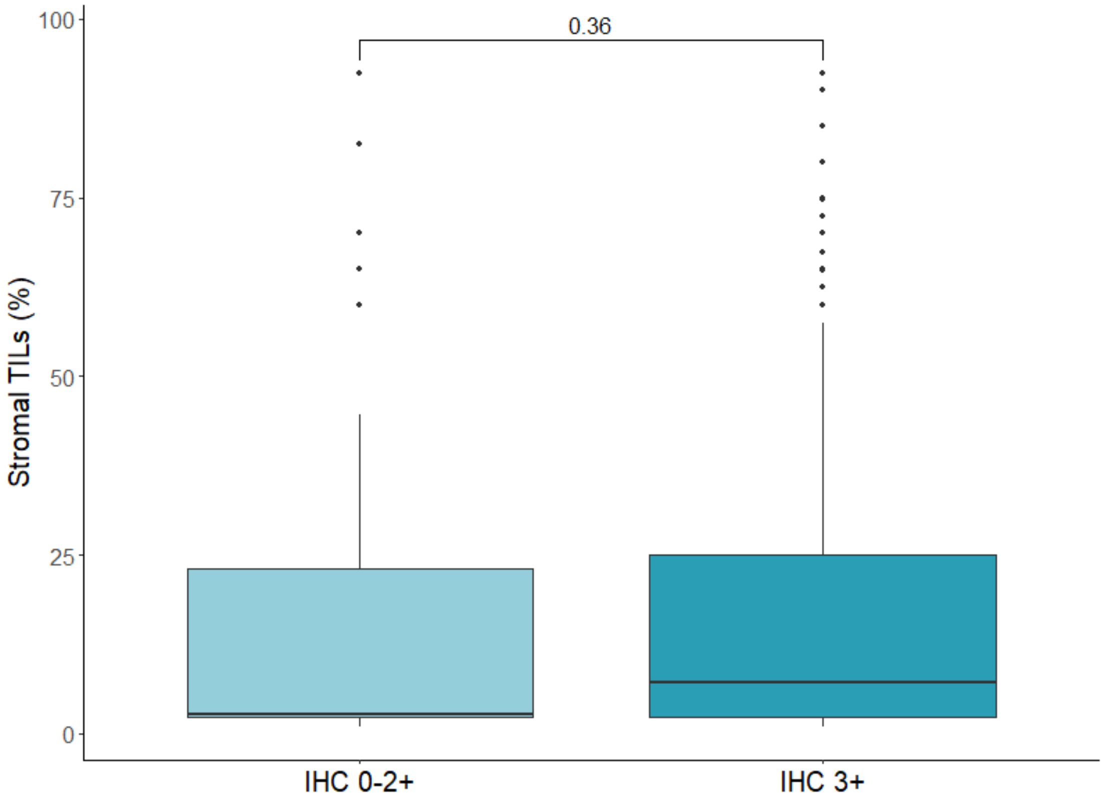

0.36
Stromal TILs (%)
100
75
50
25
0
IHC 0-2+
IHC 3+

f

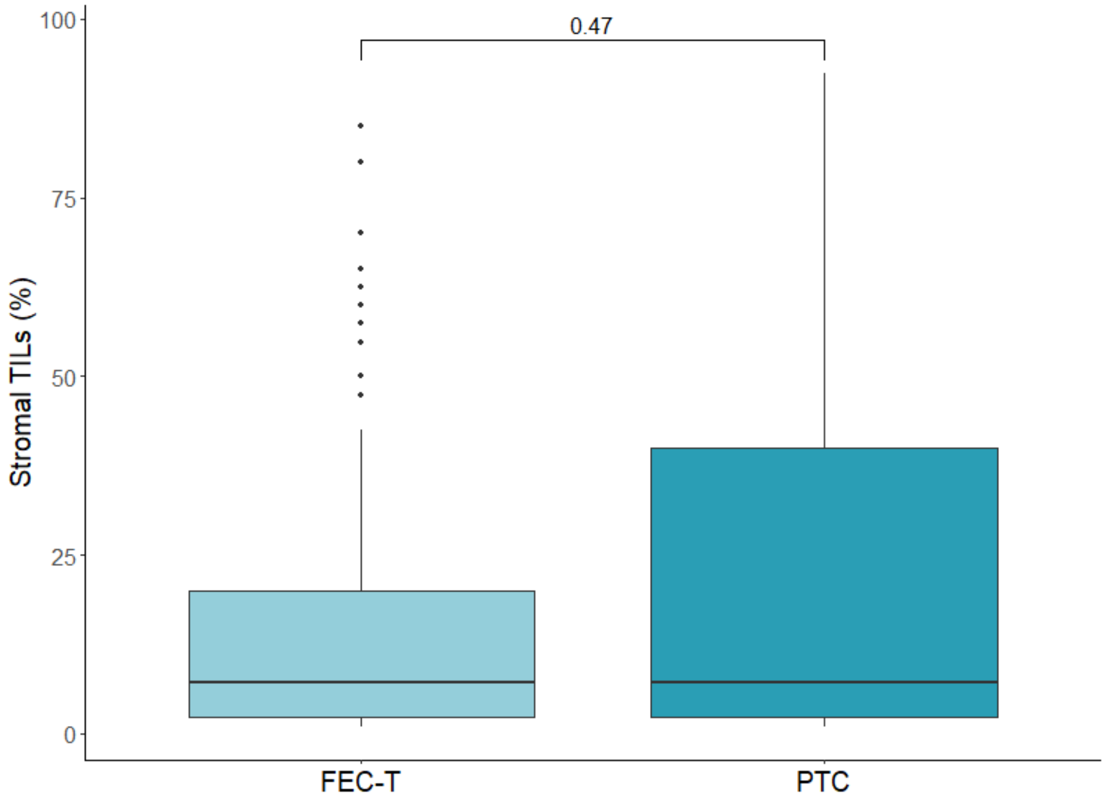
0.47
100
75
50
25
0
Stromal TILs (%)
FEC-T
PTC

g

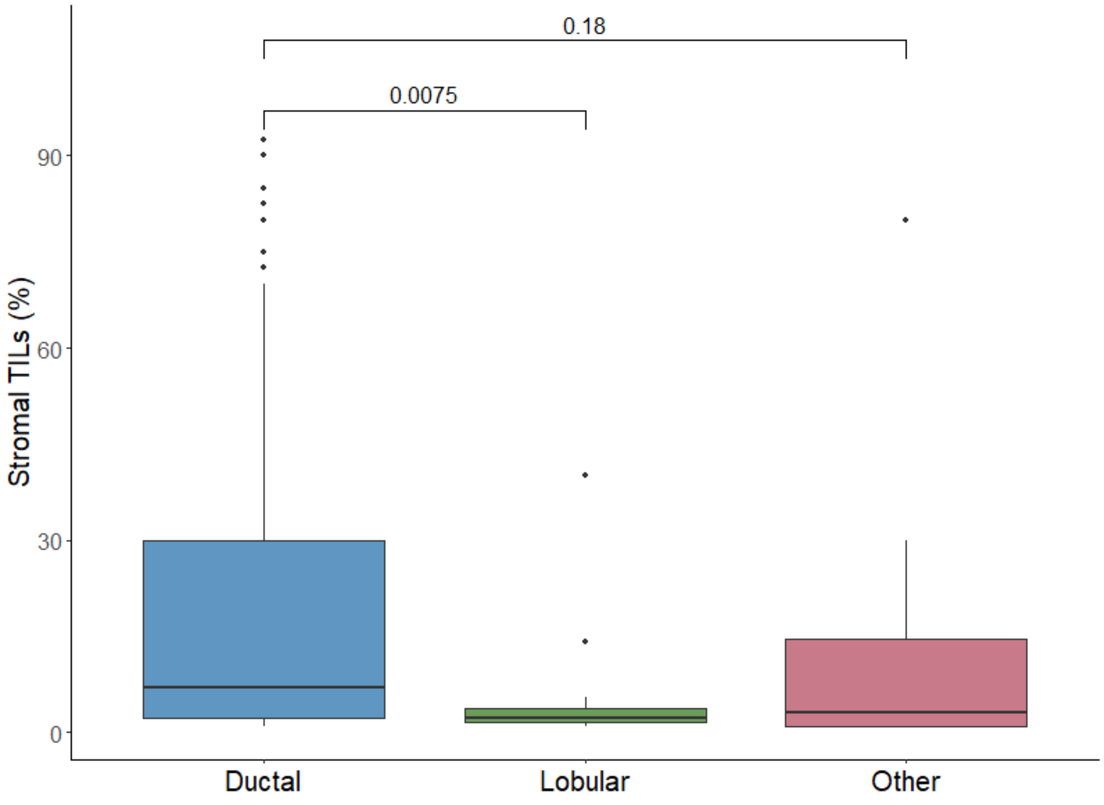
0.18
0.0075
90
60
30
0
Stromal TILs (%)
Ductal
Lobular
Other

Supplementary Figure 4. Survival curves according to TILs >14% and clinical variables

a) Kaplan-Meier curve for TILs ≤14% vs. TILs >14% at diagnosis. b) Kaplan-Meier curve for the combination of TILs (14% cut-off) and pCR after neoadjuvant treatment. c) Kaplan-Meier curve for the combination of TILs (14% cut-off) and nodal stage at diagnosis. d) Kaplan-Meier curve for the combination of TILs (14% cut-off) and hormone receptor status.
All presented p-values are from global log-rank tests.

Abbreviations: TIL = tumor infiltrating lymphocytes; IDFS = invasive disease free survival; pCR = pathological complete response; cN0 = node-negative; cN+ = node-positive; HR neg = hormone receptor negative; HR pos = hormone receptor positive

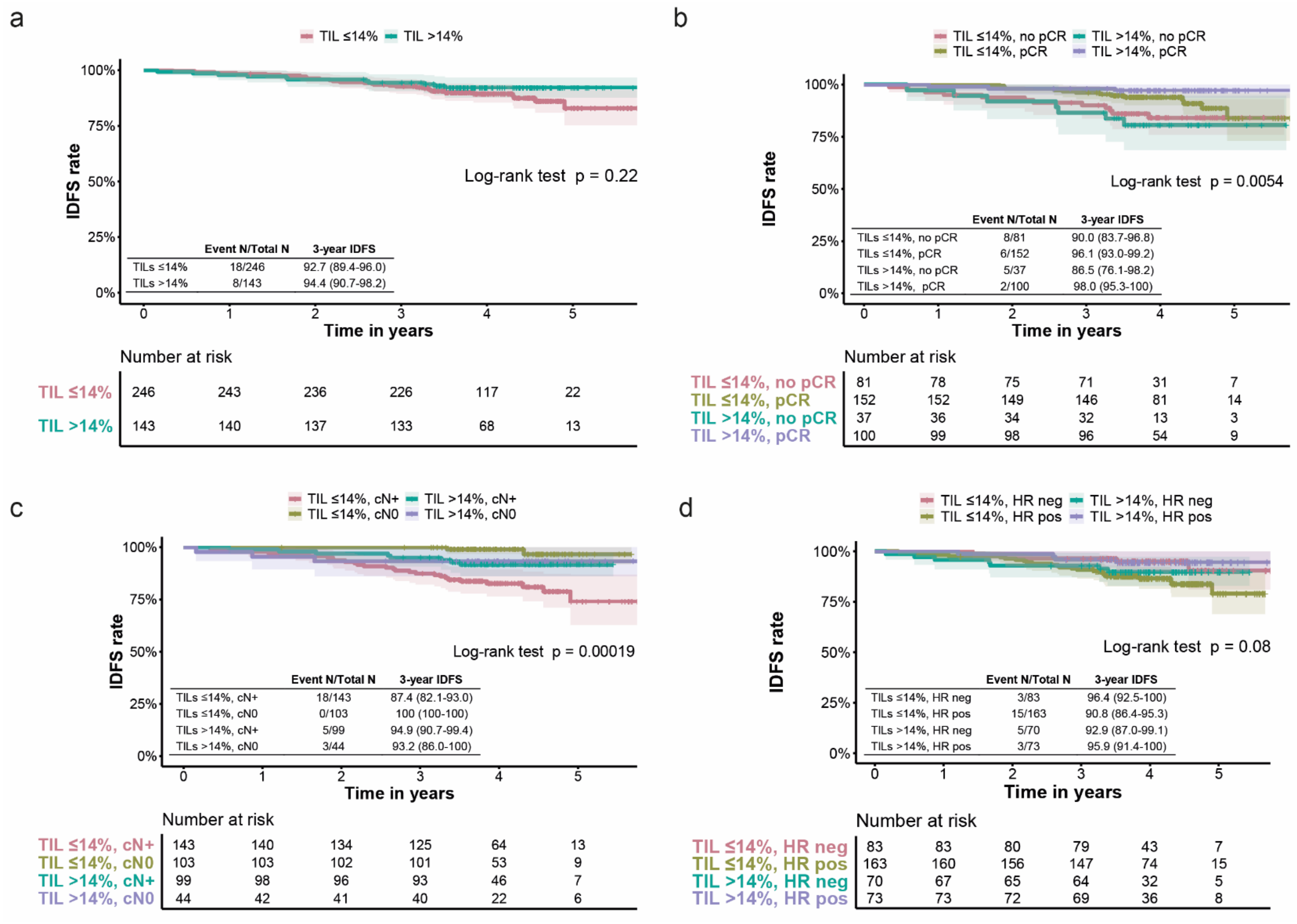

Supplementary Figure 5. Kaplan-Meier curve of IDFS per treatment arm and per TIL subgroup

a) Kaplan-Meier curve for treatment arm and TIL subgroups based on the 14% cut-off. b) a) Kaplan-Meier curve for treatment arm and TIL subgroups based on the 60% cut-off.

Abbreviations: TIL = tumor infiltrating lymphocytes; IDFS = invasive disease free survival; FEC-T = 5-Fluoruracil, Epirubicin, Cyclophosphamide, Trastuzumab, Pertuzumab; PTC = Paclitaxel, Trastuzumab, Carboplatin, Pertuzumab

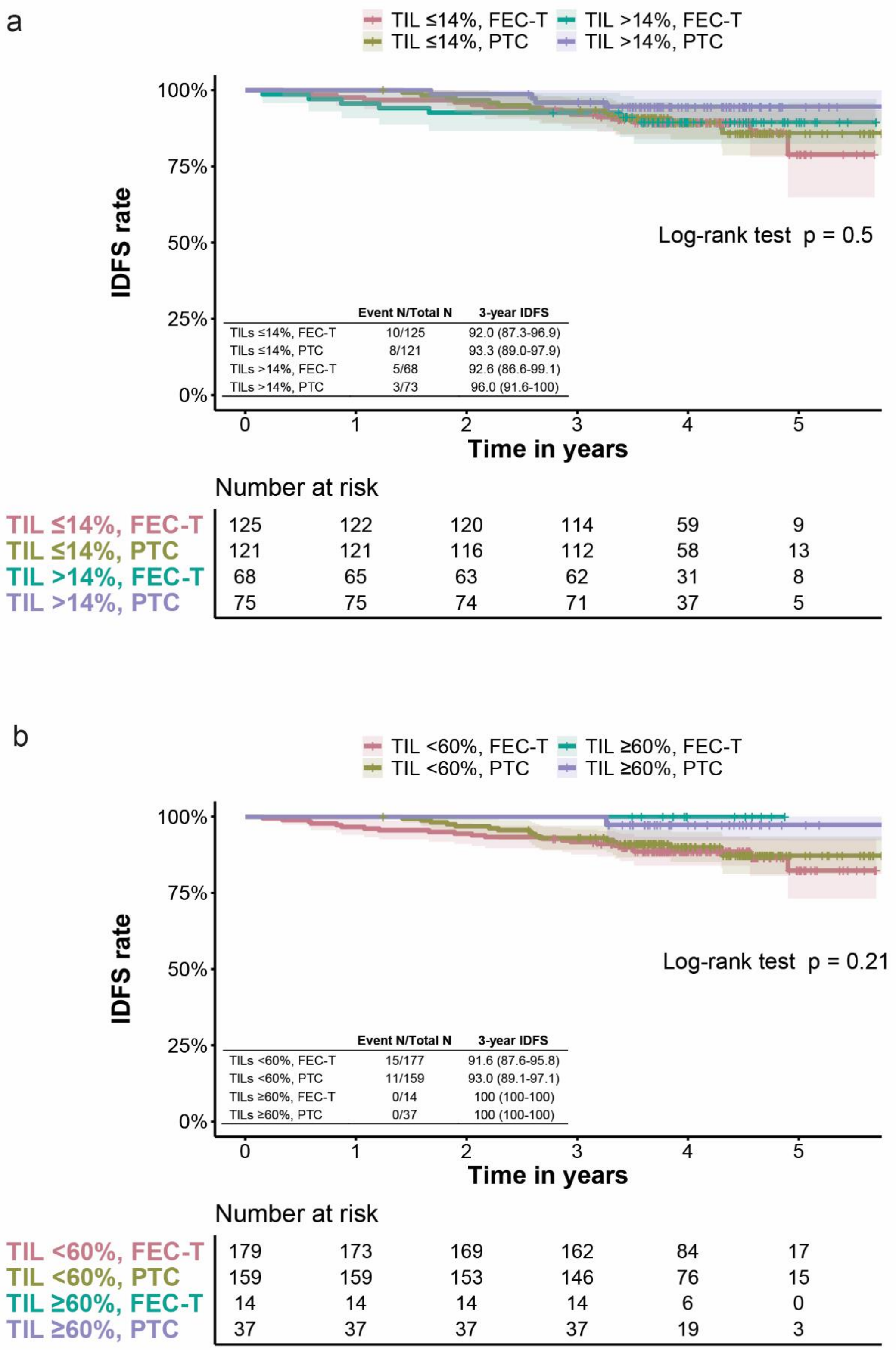
a
TIL ≤14%, FEC-T
TIL >14%, FEC-T
TIL ≤14%, PTC
TIL >14%, PTC
100%
75%
50%
25%
0%
IDFS rate
Log-rank test p = 0.5
Event N/Total N
3-year IDFS
TILs ≤14%, FEC-T 10/125 92.0 (87.3-96.9)
TILs ≤14%, PTC 8/121 93.3 (89.0-97.9)
TILs >14%, FEC-T 5/68 92.6 (86.6-99.1)
TILs >14%, PTC 3/73 96.0 (91.6-100)
0 1 2 3 4 5
Time in years
Number at risk
TIL ≤14%, FEC-T 125 122 120 114 59 9
TIL ≤14%, PTC 121 121 116 112 58 13
TIL >14%, FEC-T 68 65 63 62 31 8
TIL >14%, PTC 75 75 74 71 37 5
b
TIL <60%, FEC-T
TIL ≥60%, FEC-T
TIL <60%, PTC
TIL ≥60%, PTC
100%
75%
50%
25%
0%
IDFS rate
Log-rank test p = 0.21
Event N/Total N
3-year IDFS
TILs <60%, FEC-T 15/177 91.6 (87.6-95.8)
TILs <60%, PTC 11/159 93.0 (89.1-97.1)
TILs ≥60%, FEC-T 0/14 100 (100-100)
TILs ≥60%, PTC 0/37 100 (100-100)
0 1 2 3 4 5
Time in years
Number at risk
TIL <60%, FEC-T 179 173 169 162 84 17
TIL <60%, PTC 159 159 153 146 76 15
TIL ≥60%, FEC-T 14 14 14 14 6 0
TIL ≥60%, PTC 37 37 37 37 19 3

Supplementary Figure 6. Correlation plot of immune gene expression

Abbreviations: ER = estrogen receptor; HER2 IHC = HER2 immunohistochemistry; TIL = tumor infiltrating lymphocytes

** p <0.05*

*** p <0.01*

**** p <0.001*

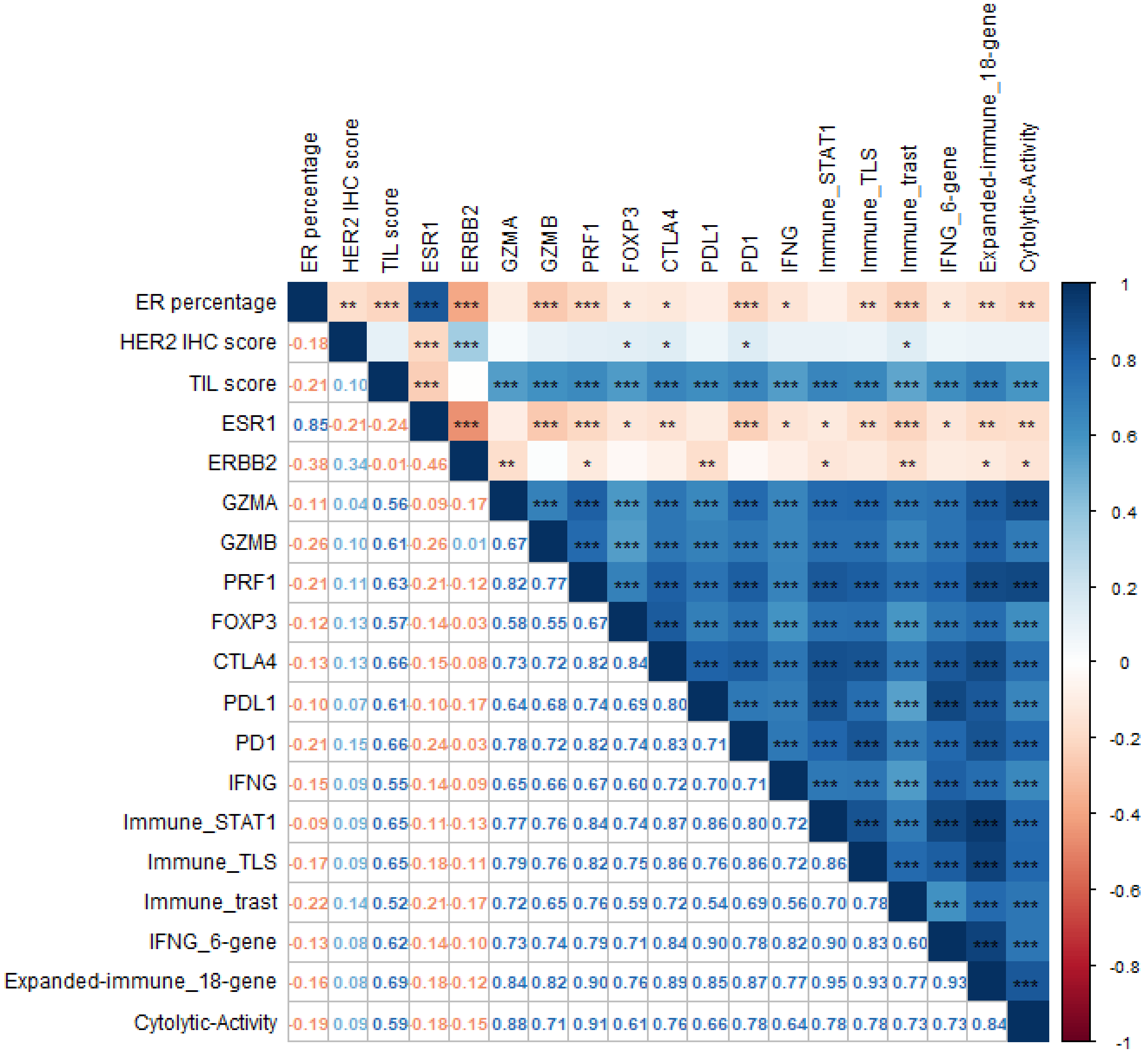

SUPPLEMENTARY TABLES

Supplementary Table 1. Percentage of concordance between TIL scores as evaluated with different cut-offs

		Scorer 2	
		**TILs ≤14%**	**TILs >14%**
**Scorer 1**	**TILs ≤14%**	217	34
	**TILs >14%**	34	97
		**TIL <60 %**	**TIL ≥60%**
	**TILs <60%**	314	28
	**TILs ≥60%**	11	29

Abbreviations: TILs = Tumor Infiltrating Lymphocytes

Supplementary Table 2. Univariable logistic regression analyses with pathological complete response as outcome

	N	Event N	OR	95% CI	p-value
**Age**	370	252	0.98	0.96, 1.00	0.089
**HR status**					
Negative	147	129	Ref	Ref	
Positive	223	123	0.17	0.10, 0.29	**<0.001**
**Grade**					
1-2	186	122	Ref	Ref	
3	167	119	1.30	0.83, 2.05	0.25
**T-stage**					
cT0-2	249	173	Ref	Ref	
cT3-4	120	78	0.82	0.52, 1.30	0.39
**N-stage**					
cN0	143	105	Ref	Ref	
cN+	227	147	0.67	0.42, 1.05	0.082
**HER2 IHC**					
1-2+	36	13	Ref	Ref	
3+	331	237	4.46	2.20, 9.41	**<0.001**
**Histology**					
Ductal	334	231	Ref	Ref	
Lobular	15	4	0.16	0.04, 0.49	**0.002**
Other	21	17	1.90	0.68, 6.71	0.26
**Treatment Arm**					
FEC-T	186	128	Ref	Ref	
PTC	184	124	0.94	0.60, 1.45	0.77
**TILs (continuous, per 10% increase)**	370	252	1.12	1.02, 1.24	**0.028**
**TILs**					
≤14%	233	152	Ref	Ref	
>14%	137	100	1.44	0.91, 2.31	0.12
**TILs**					
<10%	223	144	Ref	Ref	
11-59%	97	69	1.35	0.81, 2.29	0.25
≥60%	50	39	1.95	0.97, 4.18	0.071

Abbreviations: OR = Odds Ratio; 95% CI = 95% Confidence Interval; Ref = reference; HR status = Hormone Receptor status; cN0 = clinical node-negative; cN+ = clinical node-positive; HER2 IHC = HER2 immunohistochemistry score; FEC-T = 5-Fluoruracil, Epirubicin, Cyclophosphamide, Trastuzumab, Pertuzumab; PTC = Paclitaxel, Trastuzumab, Carboplatin, Pertuzumab; TILs = Tumor Infiltrating Lymphocytes

Supplementary Table 3. Multivariable logistic regression analyses with pathological complete response as outcome and TILs as a continuous variable

	N	Event N	aOR	95% CI	p-value
**Intercept**	349	238	10.3	1.86, 60.0	**0.008**
**Age**	349	238	0.98	0.95, 1.00	0.059
**HR status**					
Negative	142	124	Ref	Ref	
Positive	207	114	0.18	0.10, 0.32	**<0.001**
**Grade**					
1-2	184	121	Ref	Ref	
3	165	117	0.97	0.58, 1.63	0.92
**T-stage**					
cT0-2	236	162	Ref	Ref	
cT3-4	113	76	0.97	0.56, 1.69	0.92
**N-stage**					
cN0	134	100	Ref	Ref	
cN+	215	138	0.44	0.25, 0.75	**0.003**
**HER2 IHC**					
1-2+	34	13	Ref	Ref	
3+	315	225	4.35	1.94, 10.2	**<0.001**
**Treatment Arm**					
FEC-T	175	121	Ref	Ref	
PTC	174	117	0.84	0.50, 1.39	0.50
**TILs (continuous, per 10% increase)**	349	238	1.09	0.97, 1.23	0.14

Abbreviations: aOR = Adjusted Odds Ratio; 95% CI = 95% Confidence Interval; Ref = reference; HR status = Hormone Receptor status; cN0 = clinical node-negative; cN+ = clinical node-positive; HER2 IHC = HER2 immunohistochemistry score; TILs = Tumor Infiltrating Lymphocytes; FEC-T = 5-Fluoruracil, Epirubicin, Cyclophosphamide, Trastuzumab, Pertuzumab; PTC = Paclitaxel, Trastuzumab, Carboplatin, Pertuzumab

Supplementary Table 4. Multivariable logistic regression in patients with TILs ≤14% and TILs >14%

	TIL ≤14%					TIL >14%				
	**N**	**Event N**	**aOR**	**95% CI**	**p-value**	**N**	**Event N**	**aOR**	**95% CI**	**p-value**
**Age**	221	145	0.97	0.94, 1.00	0.094	128	93	0.97	0.93, 1.02	0.26
**HR status**										
Negative	78	68	Ref	Ref		64	56	Ref	Ref	
Positive	143	77	0.17	0.07, 0.35	**<0.001**	64	37	0.19	0.07, 0.46	**<0.001**
**Grade**										
1-2	130	82	Ref	Ref		54	39	Ref	Ref	
3	91	63	1.06	0.56, 2.03	0.86	74	54	0.81	0.33, 1.97	0.65
**T-stage**										
cT0-2	154	103	Ref	Ref		82	59	Ref	Ref	
cT3-4	67	42	0.90	0.45, 1.82	0.76	46	34	1.02	0.41, 2.57	0.97
**N-stage**										
cN0	97	69	Ref	Ref		37	31	Ref	Ref	
cN+	124	76	0.47	0.23, 0.92	**0.030**	91	62	0.34	0.11, 0.94	**0.048**
**HER2 IHC**										
1-2+	23	7	Ref	Ref		11	6	Ref	Ref	
3+	198	138	5.91	2.14, 18.1	**<0.001**	117	87	2.20	0.51, 9.46	0.28
**Treatment Arm**										
FEC-T	115	79	Ref	Ref		60	42	Ref	Ref	
PTC	106	66	0.73	0.39, 1.35	0.32	68	51	1.32	0.55, 3.21	0.54

Abbreviations: aOR = Adjusted Odds Ratio; 95% CI = 95% Confidence Interval; Ref = reference; HR status = Hormone Receptor status; cN0 = clinical node-negative; cN+ = clinical node-positive; HER2 IHC = HER2 immunohistochemistry score; FEC-T = 5-Fluoruracil, Epirubicin, Cyclophosphamide, Trastuzumab, Pertuzumab; PTC = Paclitaxel, Trastuzumab, Carboplatin, Pertuzumab

Supplementary Table 5. Multivariable logistic regression in patients with TILs <60% and TILs ≥60%

	TIL <60%					TIL ≥60%				
	N	Event N	aOR	95% CI	p-value	N	Event N	aOR	95% CI	p-value
**Age**	301	200	0.97	0.95, 1.00	0.064	48	38	0.96	0.87, 1.05	0.43
**HR status**										
Negative	116	102	—	—		26	22	—	—	
Positive	185	98	0.15	0.08, 0.29	**<0.001**	22	16	0.43	0.09, 1.88	0.27
**Grade**										
1-2	171	110	—	—		13	11	—	—	
3	130	90	1.03	0.59, 1.78	0.92	35	27	0.57	0.07, 2.98	0.53
**T-stage**										
cT0-2	198	132	—	—		38	30	—	—	
cT3-4	103	68	0.98	0.55, 1.78	0.96	10	8	0.80	0.12, 7.11	0.83
**N-stage**										
cN0	118	86	—	—		16	14	—	—	
cN+	183	114	0.45	0.24, 0.80	**0.008**	32	24	0.40	0.05, 2.03	0.31
**HER2 IHC**										
1-2+	28	9	—	—		6	4	—	—	
3+	273	191	5.15	2.08, 13.8	**<0.001**	42	34	2.03	0.22, 15.1	0.49
**Treatment Arm**										
FEC-T	163	111	—	—		12	10	—	—	
PTC	138	89	0.78	0.46, 1.34	0.38	36	28	0.90	0.10, 5.74	0.92

Abbreviations: aHR = Adjusted Hazard Ratio; 95% CI = 95% Confidence Interval; Ref = reference; HR status = Hormone Receptor status; cN0 = clinical node-negative; cN+ = clinical node-positive; HER2 IHC = HER2 immunohistochemistry score; FEC-T = 5-Fluoruracil, Epirubicin, Cyclophosphamide, Trastuzumab, Pertuzumab; PTC = Paclitaxel, Trastuzumab, Carboplatin, Pertuzumab

Supplementary Table 6. Multivariable logistic regression in subgroups based on hormone receptor status or nodal stage

Subgroup	Variable	N	Event N	aOR	95% CI	p-value
**HR positive**	**TILs (continuous, per 10% increase)**	207	114	1.15	0.99, 1.34	0.070
	**TILs**					
	≤14%	143	77	Ref	Ref	
	>14%	64	37	1.29	0.68, 2.50	0.44
	**TILs**					
	<60%	185	98	—	—	
	≥60%	22	16	2.90	1.00, 9.71	0.063
**HR negative**	**TIL (continuous, per 10% increase)**	142	123	0.99	0.82, 1.23	0.96
	**TILs**					
	≤14%	78	68	Ref	Ref	
	>14%	64	56	1.15	0.40, 3.42	0.80
	**TILs**					
	<60%	122	11	—	—	
	≥60%	26	1	0.78	0.10, 6.35	0.82
**cN0**	**TILs (continuous, per 10% increase)**	134	100	1.07	0.88, 136	0.54
	**TILs**					
	≤14%	97	69	Ref	Ref	
	>14%	37	31	1,62	0.57, 5.19	0.39
	**TILs**					
	<60%	118	86	—	—	
	≥60%	16	14	2.53	0.57, 18.5	0.28
**cN+**	**TILs (continuous, per 10% increase)**	215	138	1.09	0.95, 126	0.21
	**TILs**					
	≤14%	124	76	Ref	Ref	
	>14%	91	62	1.03	0.54, 1.99	0.92
	**TILs**					
	<60%	183	114	—	—	
	≥60%	32	24	1.68	0.63, 4.88	0.32

Abbreviations: aOR = Adjusted Odds Ratio; 95% CI = 95% Confidence Interval; Ref = reference; HR status = Hormone Receptor status; cN0 = clinical node-negative; cN+ = clinical node-positive

Supplementary Table 7. Pairwise log-rank tests for survival analyses of TILs combined with pathological complete response, nodal status, hormone receptor status and treatment arm

	**TILs ≤14%, no pCR**	**TILs ≤14%, pCR**	**TILs >14%, no pCR**
**TILs ≤14%, pCR**	0.1205	-	-
**TILs >14%, no pCR**	0.5929	0.0773	-
**TILs >14%, pCR**	0.0105	0.1205	0.0076
	**TILs ≤14%, cN+**	**TILs ≤14%, cN0**	**TILs >14%, cN+**
**TILs ≤14%, cN0**	0.004	-	-
**TILs >14%, cN+**	0.0718	0.0890	-
**TILs >14%, cN0**	0.1045	0.1604	0.8186
	**TILs ≤14%, HR neg**	**TILs ≤14%, HR pos**	**TILs >14%, HR neg**
**TILs ≤14%, HR pos**	0.15	-	-
**TILs >14%, HR neg**	0.46	0.46	-
**TILs >14%, HR pos**	0.85	0.15	0.46
	**TILs ≤14%, FEC-T**	**TILs ≤14%, PTC**	**TILs >14%, FEC-T**
**TILs ≤14%, PTC**	0.83	-	-
**TILs >14%, FEC-T**	0.83	0.83	-
**TILs >14%, PTC**	0.47	0.47	0.54
	**TILs <60%, no pCR**	**TILs <60%, pCR**	**TILs ≥60%, no pCR**
**TILs <60%, pCR**	0.021	-	-
**TILs ≥60%, no pCR**	0.579	0.794	-
**TILs ≥60%, pCR**	0.021	0.131	0.119
	**TILs <60%, cN+**	**TILs <60%, cN0**	**TILs ≥60%, cN+**
**TILs <60%, cN0**	0.0042	-	-
**TILs ≥60%, cN+**	0.1481	0.8047	-
**TILs ≥60%, cN0**	0.1481	0.5522	0.5522
	**TILs <60%, HR neg**	**TILs <60%, HR pos**	**TILs ≥60%, HR neg**
**TILs <60%, HR pos**	0.34	-	-
**TILs ≥60%, HR neg**	0.42	0.34	-
**TILs ≥60%, HR pos**	0.34	0.34	0.39
	**TILs <60%, FEC-T**	**TILs <60%, PTC**	**TILs ≥60%, FEC-T**
**TILs <60%, PTC**	0.62	-	-
**TILs ≥60%, FEC-T**	0.30	0.30	-
**TILs ≥60%, PTC**	0.30	0.30	0.62

Abbreviations: TILs = Tumor Infiltrating Lymphocytes; pCR = pathological complete response; cN0 = clinical node-negative; cN+ = clinical node-positive; HR = hormone receptor; FEC-T = 5-Fluoruracil, Epirubicin, Cyclophosphamide, Trastuzumab, Pertuzumab; PTC = Paclitaxel, Trastuzumab, Carboplatin, Pertuzumab; pCR = pathological complete response; TILs = Tumor Infiltrating Lymphocytes

Supplementary Table 8. Univariable Cox regression analyses with invasive disease free survival as outcome

	N	Event N	HR	95% CI	p-value
**Age**	389	40	1.02	0.99, 1.05	0.2
**HR status**					
Negative	153	12	Ref	Ref	
Positive	236	28	1.50	0.76, 2.95	0.2
**Grade**					
1-2	199	24	Ref	Ref	
3	173	14	0.64	0.33, 1.23	0.2
**T-stage**					
cT0-2	264	22	Ref	Ref	
cT3-4	124	18	1.82	0.98, 3.40	0.059
**N-stage**					
cN0	147	5	Ref	Ref	
cN+	242	35	4.46	1.75, 11.4	**0.002**
**HER2 IHC**					
1-2+	36	4	Ref	Ref	
3+	349	36	0.91	0.32, 2.56	0.9
**Treatment Arm**					
FEC-T	193	22	Ref	Ref	
PTC	196	18	0.79	0.42, 1.47	0.5
**pCR**					
No	118	19	Ref	Ref	
Yes	252	16	0.36	0.18, 0.69	**0.002**
**TILs (continuous, per 10% increase)**	389	40	0.83	0.69, 1.00	**0.049**
**TILs**					
≤14%	246	29	Ref	Ref	
>14%	143	11	0.65	0.32, 1.30	0.2
**TILs**					
<10%	236	29	—	—	
11-59%	102	10	0.80	0.39, 1.65	0.6
≥60%	51	1	0.15	0.02, 1.13	0.065

Abbreviations: HR = Hazard Ratio; 95% CI = 95% Confidence Interval; Ref = reference; HR status = Hormone Receptor status; cN0 = clinical node-negative; cN+ = clinical node-positive; HER2 IHC = HER2 immunohistochemistry score; FEC-T = 5-Fluoruracil, Epirubicin, Cyclophosphamide, Trastuzumab, Pertuzumab; PTC = Paclitaxel, Trastuzumab, Carboplatin, Pertuzumab; pCR = pathological complete response; TILs = Tumor Infiltrating Lymphocytes

Supplementary Table 9. Multivariable Cox regression analyses with invasive disease free survival as outcome and TILs as a continuous variable

	N	Event N	aHR	95% CI	p-value
**Age**	367	38	1.03	1.00, 1.07	0.088
**HR status**					
Negative	148	12	Ref	Ref	
Positive	219	26	1.23	0.61, 2.49	0.56
**Grade**					
1-2	197	24	Ref	Ref	
3	170	14	0.77	0.39, 1.51	0.45
**T-stage**					
cT0-2	250	22	Ref	Ref	
cT3-4	117	16	1.30	0.67, 2.51	0.44
**N-stage**					
cN0	138	5	Ref	Ref	
cN+	229	33	4.57	1.74, 12.0	**0.002**
**HER2 IHC**					
1-2+	34	3	Ref	Ref	
3+	333	35	0.96	0.29, 3.19	0.95
**Treatment Arm**					
FEC-T	182	22	Ref	Ref	
PTC	185	16	0.72	0.38, 1.37	0.31
**TILs (continuous, per 10% increase)**	367	38	0.86	0.70, 1.04	0.12

Abbreviations: aHR = Adjusted Hazard Ratio; 95% CI = 95% Confidence Interval; Ref = reference; HR status = Hormone Receptor status; cN0 = clinical node-negative; cN+ = clinical node-positive; HER2 IHC = HER2 immunohistochemistry score; FEC-T = 5-Fluoruracil, Epirubicin, Cyclophosphamide, Trastuzumab, Pertuzumab; PTC = Paclitaxel, Trastuzumab, Carboplatin, Pertuzumab; TILs = Tumor Infiltrating Lymphocytes

Supplementary Table 10. Multivariable Cox regression in TIL subgroups based on the 14% cut-off with invasive disease free survival as outcome

	TILs ≤14%					TILs >14%				
	N	Event N	aHR	95% CI	p-value	N	Event N	aHR	95% CI	p-value
**Age**	233	27	1.04	1.00, 1.08	0.051	134	11	1.01	0.95, 1.08	0.73
**HR status**										
Negative	81	5	Ref	Ref		67	7	Ref	Ref	
Positive	152	22	2.14	0.80, 5.73	0.131	67	4	0.47	0.13, 1.66	0.24
**Grade**										
1-2	139	18	Ref	Ref		58	6	Ref	Ref	
3	94	9	0.69	0.30, 1.57	0.372	76	5	0.57	0.16, 1.97	0.37
**T-stage**										
cT0-2	162	16	Ref	Ref		88	6	Ref	Ref	
cT3-4	71	11	1.07	0.48, 2.36	0.872	46	5	1.48	0.44, 4.94	0.53
**N-stage**										
cN0	99	2	Ref	Ref		39	3	Ref	Ref	
cN+	134	25	10.7	2.43, 47.2	**0.002**	95	8	1.15	0.30, 4.41	0.84
**HER2 IHC**										
1-2+	23	2	Ref	Ref		11	1	Ref	Ref	
3+	210	25	0.93	0.22, 4.06	0.928	123	10	0.66	0.08, 5.44	0.70
**Treatment Arm**										
FEC-T	119	15	Ref	Ref		63	7	Ref	Ref	
PTC	114	12	0.75	0.35, 1.61	0.457	71	4	0.48	0.14, 1.69	0.25

Abbreviations: aHR = Adjusted Hazard Ratio; 95% CI = 95% Confidence Interval; Ref = reference; HR status = Hormone Receptor status; cN0 = clinical node-negative; cN+ = clinical node-positive; HER2 IHC = HER2 immunohistochemistry score; FEC-T = 5-Fluoruracil, Epirubicin, Cyclophosphamide, Trastuzumab, Pertuzumab; PTC = Paclitaxel, Trastuzumab, Carboplatin, Pertuzumab

Supplementary Table 11. Multivariable Cox regression in TIL subgroups based on the 60% cut-off with invasive disease free survival as outcome

	TILs <60%					TILs ≥60%				
	**N**	**Event N**	**aHR**	**95% CI**	**p-value**	**N**	**Event N**	**aHR**	**95% CI**	**p-value**
**Age**	318	37	1.03	1.00, 1.07	0.072	49	1	1.06	0.71, 1.60	0.78
**HR status**										
Negative	122	11	—	—		26	1	—	—	
Positive	196	26	1.36	0.66, 2.79	0.40	23	0	0.00	0.00, Inf	>0.99
**Grade**										
1-2	184	24	—	—		13	0	—	—	
3	134	13	0.75	0.38, 1.48	0.40	36	1	518,038,825	0.00, Inf	>0.99
**T-stage**										
cT0-2	211	21	—	—		39	1	—	—	
cT3-4	107	16	1.31	0.67, 2.54	0.43	10	0	0.31	0.00, Inf	>0.99
**N-stage**										
cN0	121	5	—	—		17	0	—	—	
cN+	197	32	4.23	1.61, 11.2	**0.004**	32	1	690,480,756	0.00, Inf	>0.99
**HER2 IHC**										
1-2+	28	3	—	—		6	0	—	—	
3+	290	34	0.92	0.28, 3.06	0.90	43	1	746,461,606	0.00, Inf	>0.99
**Treatment Arm**										
FEC-T	169	22	—	—		13	0	—	—	
PTC	149	15	0.73	0.38, 1.41	0.35	36	1	519,621,254	0.00, Inf	>0.99

Abbreviations: aHR = Adjusted Hazard Ratio; 95% CI = 95% Confidence Interval; Ref = reference; HR status = Hormone Receptor status; cN0 = clinical node-negative; cN+ = clinical node-positive; HER2 IHC = HER2 immunohistochemistry score; FEC-T = 5-Fluoruracil, Epirubicin, Cyclophosphamide, Trastuzumab, Pertuzumab; PTC = Paclitaxel, Trastuzumab, Carboplatin, Pertuzuma

Supplementary Table 12. Multivariable Cox regression in subgroups based on hormone receptor status or nodal stage with invasive disease free survival as outcome

Subgroup	Variable	N	Event N	aHR	95% CI	p-value
**HR positive**	**TILs (continuous, per 10% increase)**	219	26	0.71	0.50, 1.01	0.058
	**TILs**					
	≤14%	152	22	Ref	Ref	
	>14%	67	4	0.34	0.12, 1.00	**0.050**
	**TILs**					
	<60%	196	26	—	—	
	≥60%	23	0	0.00	0.00, Inf	>0.99
**HR negative**	**TIL (continuous, per 10% increase)**	148	12	1.04	0.80, 1.34	0.78
	**TILs**					
	≤14%	81	5	Ref	Ref	
	>14%	67	7	1.97	0.61, 6.32	0.25
	**TILs**					
	<60%	122	11	—	—	
	≥60%	26	1	0.78	0.10, 6.35	0.82
**cN0**	**TILs (continuous, per 10% increase)**	138	5	1.04	0.67, 1.60	0.88
	**TILs**					
	≤14%	99	2	Ref	Ref	
	>14%	39	3	4.5	0.66, 30.6	0.42
	**TILs**					
	<60%	121	5	—	—	
	≥60%	17	0	0.00	0.00, Inf	>0.99
**cN+**	**TILs (continuous, per 10% increase)**	229	33	0.83	0.67, 1.03	0.091
	**TILs**					
	≤14%	134	25	Ref	Ref	
	>14%	95	8	0.51	0.23, 1.15	0.11
	**TILs**					
	<60%	197	32	—	—	
	≥60%	32	1	0.24	0.03, 1.81	0.17

Abbreviations: aHR = Adjusted Hazard Ratio; 95% CI = 95% Confidence Interval; Ref = reference; HR status = Hormone Receptor status; cN0 = clinical node-negative; cN+ = clinical node-positive

Supplementary Table 13. Univariable and multivariable logistic regression analyses of genes and gene signatures with pCR

	Univariable analysis					Multivariable analysis				
	N	OR	95% CI	p-value	q-value	N	aOR	95% CI	p-value	q-value
**ESR1**	306	0.24	0.15, 0.37	5.75e-10	**4.60e-09**	287	0.38	0.18,0.78	9.15e-03	7.32e-02
**ERBB2**	306	8.26	4.63, 15.6	7.71e-12	**1.23e-10**	287	11.56	4.98,29.41	5.72e-08	**9.15e-07**
**immune_STAT1**	306	1.51	0.91, 2.52	1.12e-01	1.36e-01	287	1.05	0.49,2.24	9.04e-01	9.04e-01
**immune_TLS**	306	1.66	0.99, 2.81	5.56e-02	8.90e-02	287	1.35	0.63,2.93	4.43e-01	8.71e-01
**immune_trastuzumab**	306	1.82	1.08, 3.11	2.58e-02	5.90e-02	287	1.29	0.63,2.66	4.9e-01	8.71e-01
**GZMA**	306	1.16	0.66, 2.04	6.07e-01	6.07e-01	287	0.86	0.39,1.89	7.07e-01	9.04e-01
**GZMB**	306	2.52	1.53, 4.23	3.56e-04	**1.90e-03**	287	2.12	1.07,4.28	3.23e-02	1.72e-01
**PRF1**	306	2.20	1.20, 4.12	1.21e-02	**3.76e-02**	287	1.40	0.57,3.46	4.62e-01	8.71e-01
**FOXP3**	306	1.41	0.87, 2.28	1.59e-01	1.70e-01	287	1.05	0.55,1.98	8.8e-01	9.04e-01
**CTLA4**	306	1.54	0.93, 2.57	9.38e-02	1.25e-01	287	1.08	0.51,2.25	8.4e-01	9.04e-01
**PDL1**	306	1.50	0.90, 2.52	1.19e-01	1.36e-01	287	1.20	0.57,2.52	6.38e-01	9.04e-01
**PD1**	306	1.98	1.15, 3.43	1.41e-02	**3.76e-02**	287	1.27	0.57,2.84	5.49e-01	8.78e-01
**IFNG**	306	1.83	1.18, 2.91	8.29e-03	**3.32e-02**	287	1.55	0.84,2.90	1.65e-01	6.60e-01
**IFNG_6-gene**	306	1.69	1.04, 2.75	3.40e-02	6.79e-02	287	1.56	0.77,3.18	2.2e-01	7.04e-01
**expanded-immune_18-gene**	306	1.68	1.02, 2.79	4.16e-02	7.39e-02	287	1.31	0.61,2.86	4.9e-01	8.71e-01
**cytolytic-activity**	306	1.50	0.94, 2.44	9.28e-02	1.25e-01	287	0.96	0.49,1.89	9e-01	9.04e-01

Abbreviations: OR = Odds Ratio; aOR =Adjusted Odds Ratio; 95% CI = 95% Confidence Interval q-value?

Supplementary Table 14. Univariable and multivariable logistic regression analyses of genes and gene signatures with IDFS

	Univariable analysis					Multivariable analysis				
	N	HR	95% CI	p-value	q-value	N	aHR	95% CI	p-value	q-value
**ESR1**	323	1.01	0.55, 1.85	9.83e-01	9.83e-01	303	0.93	0.36,2.35	8.71e-01	8.83e-01
**ERBB2**	323	0.70	0.35, 1.41	3.20e-01	4.85e-01	303	0.48	0.2,1.17	1.06e-01	4.27e-01
**immune_STAT1**	323	0.69	0.32, 1.47	3.38e-01	4.85e-01	303	0.67	0.23,1.94	4.57e-01	6.65e-01
**immune_TLS**	323	0.58	0.27, 1.27	1.73e-01	4.01e-01	303	0.42	0.14,1.27	1.23e-01	4.27e-01
**immune_trastuzumab**	323	0.85	0.39, 1.85	6.78e-01	7.75e-01	303	0.89	0.33,2.4	8.23e-01	8.83e-01
**GZMA**	323	0.92	0.39, 2.18	8.46e-01	9.03e-01	303	0.92	0.3,2.81	8.83e-01	8.83e-01
**GZMB**	323	0.62	0.29, 1.29	2.00e-01	4.01e-01	303	0.56	0.21,1.49	2.47e-01	4.66e-01
**PRF1**	323	0.54	0.21, 1.35	1.88e-01	4.01e-01	303	0.50	0.15,1.68	2.59e-01	4.66e-01
**FOXP3**	323	0.61	0.31, 1.18	1.43e-01	4.01e-01	303	0.48	0.22,1.04	6.29e-02	4.27e-01
**CTLA4**	323	0.58	0.28, 1.20	1.41e-01	4.01e-01	303	0.45	0.17,1.14	9.2e-02	4.27e-01
**PDL1**	323	0.50	0.23, 1.08	7.73e-02	4.01e-01	303	0.46	0.16,1.29	1.38e-01	4.27e-01
**PD1**	323	0.64	0.28, 1.46	2.86e-01	4.85e-01	303	0.55	0.17,1.73	3.05e-01	4.88e-01
**IFNG**	323	0.85	0.44, 1.66	6.36e-01	7.75e-01	303	1.28	0.56,2.95	5.59e-01	7.45e-01
**IFNG_6-gene**	323	0.61	0.29, 1.28	1.92e-01	4.01e-01	303	0.56	0.21,1.53	2.62e-01	4.66e-01
**expanded-immune_18-gene**	323	0.59	0.28, 1.26	1.73e-01	4.01e-01	303	0.45	0.15,1.37	1.6e-01	4.27e-01
**cytolytic-activity**	323	0.71	0.34, 1.49	3.63e-01	4.85e-01	303	0.82	0.33,2.07	6.77e-01	8.33e-01

Abbreviations: HR = Hazard Ratio; aHR =Adjusted Hazard Ratio; 95% CI = 95% Confidence Interval

Supplementary Table 15. Multivariable logistic and Cox regression analyses of gene expression in the FEC-T subgroup

	pCR					IDFS				
	**N**	**aOR**	**95% CI**	**p-value**	**q-value**	**N**	**aHR**	**95% CI**	**p-value**	**q-value**
**ESR1**	141	0.46	0.15,1.23	1.34e-01	7.70e-01	146	2.07	0.5,8.62	3.17e-01	3.62e-01
**ERBB2**	141	18.34	4.62,92.47	1.23e-04	**1.97e-03**	146	0.70	0.18,2.75	6.09e-01	6.09e-01
**immune_STAT1**	141	0.46	0.12,1.64	2.43e-01	7.70e-01	146	0.17	0.03,0.89	3.55e-02	6.52e-02
**immune_TLS**	141	0.63	0.17,2.14	4.63e-01	7.70e-01	146	0.10	0.02,0.56	8.3e-03	**4.90e-02**
**immune_trastuzumab**	141	0.67	0.22,2.03	4.76e-01	7.70e-01	146	0.39	0.09,1.62	1.94e-01	2.39e-01
**GZMA**	141	0.51	0.13,1.9	3.22e-01	7.70e-01	146	0.30	0.05,1.72	1.78e-01	2.37e-01
**GZMB**	141	1.64	0.57,4.82	3.59e-01	7.70e-01	146	0.23	0.06,0.91	3.67e-02	6.52e-02
**PRF1**	141	0.73	0.17,3.08	6.73e-01	8.27e-01	146	0.17	0.03,0.88	3.43e-02	6.52e-02
**FOXP3**	141	0.79	0.28,2.09	6.41e-01	8.27e-01	146	0.15	0.05,0.48	1.37e-03	**2.19e-02**
**CTLA4**	141	0.75	0.24,2.22	6.03e-01	8.27e-01	146	0.17	0.04,0.72	1.55e-02	6.20e-02
**PDL1**	141	0.66	0.21,2.02	4.67e-01	7.70e-01	146	0.20	0.04,0.87	3.16e-02	6.52e-02
**PD1**	141	1.26	0.35,4.56	7.24e-01	8.27e-01	146	0.13	0.02,0.85	3.27e-02	6.52e-02
**IFNG**	141	0.96	0.34,2.72	9.36e-01	9.36e-01	146	1.43	0.4,5.07	5.81e-01	6.09e-01
**IFNG_6-gene**	141	0.93	0.28,3.07	8.99e-01	9.36e-01	146	0.29	0.07,1.27	9.99e-02	1.45e-01
**expanded-immune_18-gene**	141	0.62	0.16,2.3	4.81e-01	7.70e-01	146	0.11	0.02,0.58	9.19e-03	**4.90e-02**
**cytolytic-activity**	141	0.61	0.19,1.91	3.93e-01	7.70e-01	146	0.22	0.04,1.3	9.45e-02	1.45e-01

Abbreviations: HR = Hazard Ratio; aHR =Adjusted Hazard Ratio; 95% CI = 95% Confidence Interval

Supplementary Table 16. Multivariable logistic and Cox regression analyses of gene expression in the PTC subgroup

	pCR					IDFS				
	N	aOR	95% CI	p-value	q-value	N	aHR	95% CI	p-value	q-value
**ESR1**	146	0.34	0.11,0.95	4.21e-02	1.83e-01	157	0.52	0.12,2.22	3.73e-01	9.15e-01
**ERBB2**	146	8.83	2.98,30.94	2.32e-04	**3.71e-03**	157	0.37	0.12,1.12	7.84e-02	9.15e-01
**immune_STAT1**	146	1.98	0.72,5.67	1.9e-01	2.76e-01	157	1.78	0.42,7.6	4.39e-01	9.15e-01
**immune_TLS**	146	2.82	0.96,8.97	6.71e-02	1.83e-01	157	1.40	0.29,6.72	6.73e-01	9.15e-01
**immune_trastuzumab**	146	2.36	0.87,6.76	9.82e-02	1.96e-01	157	2.05	0.58,7.18	2.62e-01	9.15e-01
**GZMA**	146	1.40	0.5,4	5.22e-01	5.59e-01	157	3.19	0.61,16.8	1.71e-01	9.15e-01
**GZMB**	146	3.31	1.26,9.14	1.72e-02	1.38e-01	157	1.29	0.34,4.94	7.09e-01	9.15e-01
**PRF1**	146	2.74	0.8,10.03	1.15e-01	2.04e-01	157	1.29	0.21,8.07	7.84e-01	9.15e-01
**FOXP3**	146	1.35	0.56,3.29	5e-01	5.59e-01	157	1.50	0.34,6.52	5.9e-01	9.15e-01
**CTLA4**	146	1.72	0.6,5.14	3.14e-01	4.19e-01	157	1.10	0.2,6.03	9.15e-01	9.15e-01
**PDL1**	146	2.17	0.76,6.51	1.54e-01	2.46e-01	157	0.86	0.17,4.49	8.61e-01	9.15e-01
**PD1**	146	1.30	0.45,3.89	6.29e-01	6.29e-01	157	1.15	0.21,6.24	8.69e-01	9.15e-01
**IFNG**	146	2.26	1.02,5.23	4.83e-02	1.83e-01	157	1.21	0.35,4.15	7.65e-01	9.15e-01
**IFNG_6-gene**	146	2.37	0.95,6.16	6.88e-02	1.83e-01	157	1.09	0.26,4.62	9.04e-01	9.15e-01
**expanded-immune_18-gene**	146	2.45	0.87,7.2	9.44e-02	1.96e-01	157	1.60	0.36,7.04	5.33e-01	9.15e-01
**cytolytic-activity**	146	1.33	0.56,3.22	5.24e-01	5.59e-01	157	1.77	0.54,5.8	3.47e-01	9.15e-01

Abbreviations: HR = Hazard Ratio; aHR =Adjusted Hazard Ratio; 95% CI = 95% Confidence Interval
